# A Novel α‐Synuclein K58N Missense Variant in a Patient with Parkinson's Disease

**DOI:** 10.1002/mds.70030

**Published:** 2025-09-04

**Authors:** Mohammed Al‐Azzani, Sandrina Weber, Nagendran Ramalingam, Maria Ramón, Liana Shvachiy, Gonçalo Mestre, Michael Zech, Kevin Sicking, Alain Ibáñez de Opakua, Vidyashree Jayanthi, Leslie Amaral, Aishwarya Agarwal, Aswathy Chandran, Susana R. Chaves, Juliane Winkelmann, Claudia Trenkwalder, Maike Schwager, Silke Pauli, Ulf Dettmer, Claudio O. Fernández, Janin Lautenschläger, Markus Zweckstetter, Ruben Fernandez‐Busnadiego, Brit Mollenhauer, Tiago Fleming Outeiro

**Affiliations:** ^1^ University Medical Center Göttingen Department of Experimental Neurodegeneration, Center for Biostructural Imaging of Neurodegeneration Göttingen Germany; ^2^ Department of Neurology University Medical Center Göttingen Göttingen Germany; ^3^ Ann Romney Center for Neurologic Diseases, Brigham and Women's Hospital and Harvard Medical School Boston Massachusetts USA; ^4^ Institute of Neurogenomics, Helmholtz Zentrum München, German Research Center for Environmental Health Neuherberg Germany; ^5^ Institute of Human Genetics, TUM School of Medicine and Health, Technical University of Munich Munich Germany; ^6^ Institute for Advanced Study, Technical University of Munich Garching Germany; ^7^ University Medical Center Göttingen, Institute for Neuropathology Göttingen Germany; ^8^ Aligning Science Across Parkinson's (ASAP) Collaborative Research Network Chevy Chase Maryland USA; ^9^ German Center for Neurodegenerative Diseases Göttingen Germany; ^10^ CBMA–Centre of Molecular and Environmental Biology, School of Sciences, University of Minho Braga Portugal; ^11^ Cambridge Institute for Medical Research, University of Cambridge, Cambridge Biomedical Campus Cambridge United Kingdom; ^12^ Munich Cluster for Systems Neurology (SyNergy) Munich Germany; ^13^ German Center for Mental Health (DZPG), partner site Munich‐Augsburg Munich‐Augsburg Germany; ^14^ Department of Neurosurgery University Medical Centre Göttingen Göttingen Germany; ^15^ Paracelsus‐Elena‐Klinik Kassel Germany; ^16^ Institute of Human Genetics, University Medical Center Göttingen Göttingen Germany; ^17^ Max Planck Laboratory for Structural Biology, Chemistry and Molecular Biophysics of Rosario (MPLbioR, UNR‐MPINAT), Partner Laboratory of the Max Planck Institute for Multidisciplinary Sciences (MPINAT, MPG). Centro de Estudios Interdisciplinarios, Universidad Nacional de Rosario Rosario Argentina; ^18^ Department for NMR‐Based Structural Biology Max Planck Institute for Multidisciplinary Sciences Göttingen Germany; ^19^ Cluster of Excellence “Multiscale Bioimaging: From Molecular Machines to Networks of Excitable Cells,” University of Göttingen Göttingen Germany; ^20^ Faculty of Physics University of Göttingen Göttingen Germany; ^21^ Translational and Clinical Research Institute, Faculty of Medical Sciences, Newcastle University Newcastle Upon Tyne United Kingdom; ^22^ Max Planck Institute for Multidisciplinary Sciences Göttingen Germany

**Keywords:** α‐synuclein, Parkinson's disease, genetics, neurodegeneration, protein aggregation

## Abstract

**Background:**

Parkinson's disease (PD) is a complex multifactorial disorder with a genetic component in about 15% of cases. Multiplications and point mutations in *SNCA* gene, encoding α‐synuclein (aSyn), are linked to rare familial forms of PD.

**Objective:**

Our goal was to assess the clinical presentation and the biological effects of a novel K58N aSyn mutation identified in a patient with PD.

**Methods:**

We describe the clinical presentation associated with the novel mutation, together with genetic testing through whole exome sequencing (WES). Furthermore, we conducted extensive biophysical and cellular assays to assess the functional consequences of this novel variant.

**Results:**

The patient exhibited typical features of sporadic PD with early onset and a benign disease course. WES showed a novel heterozygous missense variant in *SNCA* (NM_000345.4, c.174G>C; p.K58N). A positive family history of PD was evident, because both a parent and a grandparent had been diagnosed with PD but were deceased. The patient underwent deep brain stimulation surgery 13 years postdiagnosis, showing stable, long‐term improvements in motor symptoms. Biophysical studies demonstrated K58N substitution causes local structural effects, disrupts membrane binding, and enhances aSyn in vitro aggregation. In cellular systems, K58N aSyn produces fewer inclusions per cell and does not form condensates. The variant increases aSyn cytoplasmic distribution and displays aberrant activity‐dependent dynamic serine‐129 phosphorylation.

**Conclusions:**

The clinical presentation associated with the novel K58N aSyn mutation suggests a relatively benign PD course consistent with the phenotypic spectrum of idiopathic PD. Overall, our molecular studies provide novel insight into the biology and pathobiology of aSyn. © 2025 The Author(s). *Movement Disorders* published by Wiley Periodicals LLC on behalf of International Parkinson and Movement Disorder Society.

Parkinson's disease (PD) is an age‐associated neurodegenerative disorder classically manifesting with cardinal motor symptoms of tremor, rigidity, and bradykinesia, alongside nonmotor symptoms such as depression, autonomic dysfunction, and dementia.^1^, ^2^ Neuropathologically, PD involves progressive dopaminergic neuron degeneration in the substantia nigra and Lewy bodies (LBs), intraneuronal inclusions predominantly composed of aggregated α‐synuclein (aSyn).^3^, ^4^, ^5^, ^6^ aSyn misfolding and aggregation occur in other synucleinopathies, such as dementia with LBs (DLB) and multiple system atrophy (MSA), with increasing evidence for distinct aSyn fibril structures within inclusions across synucleinopathies.^7^, ^8^, ^9^

aSyn, an intrinsically disordered protein, represents a significant focus of neuroscience research because of its central role in PD and related synucleinopathies, and its abundance and function in the nervous system. It is primarily concentrated in presynaptic terminals of neurons but is also present in other cellular compartments, including the nucleus, where it can interact with membranes adopting helical conformations and tetrameric structures.^10^, ^11^ Nevertheless, its precise physiological functions remain poorly understood, especially in nonneuronal cells.

In pathological conditions, aSyn misfolds and aggregates, disrupting cellular homeostasis and potentially causing neuronal death. Dopaminergic neurons in the substantia nigra are particularly vulnerable to these aggregates, although underlying mechanisms remain unclear. Although aSyn aggregation is often seen as the main driver of neurodegeneration, other theories suggest loss of functional monomeric aSyn because of aggregation (synucleinopenia) may contribute.^12^, ^13^ In reality, both proteinopathy and proteinopenia likely contribute to disease.

Pathogenic missense variants in *SNCA*, which encodes aSyn, cause familial PD forms, highlighting the importance of understanding their molecular effects to elucidate disease mechanisms. In fact, since the discovery of the first *SNCA* variant (A53T),^3^ PD genetics has identified multiple additional *SNCA* variants and variants in other PD‐related genes.^14^, ^15^ These aSyn variants include p.G14R, p.V15A, p.A30G, p.A30P, p.E46K, p.H50Q, p.G51D, p.A53T, p.A53E, p.A53V, and p.T72M, highlighting aSyn's central role in PD.^3^, ^6^, ^16^, ^17^, ^18^, ^19^, ^20^, ^21^, ^22^, ^23^, ^24^ However, pathogenic SNCA variants are extremely rare, accounting for only 0.1% to 0.2% of cases.^14^ SNCA duplications or triplications also cause familial PD.^25^ SNCA triplications show a dose‐dependent effect, causing early‐onset, fully penetrant PD with severe nonmotor symptoms like depression, psychosis, and cognitive decline,^26^, ^27^ whereas duplications have variable presentations and are more often associated with DLB than PD.^26^ Missense variants are generally rarer than duplications^15^, ^28^ and show a broad clinical spectrum, from idiopathic PD‐like to atypical neurodegenerative phenotypes.^29^ Notably, several heterozygous *SNCA* missense variants have been associated with milder clinical courses. The p.A30P and p.A30G substitutions often cause tremor‐predominant PD with slow progression and long‐preserved cognition, resembling idiopathic forms of PD.^6^, ^22^ Similarly, the p.H50Q variant shows reduced penetrance, with many heterozygous carriers remaining asymptomatic or developing mild, late‐onset symptoms. These findings suggest that not all *SNCA* missense variants lead to a severe disease course, underscoring the importance of careful phenotypic documentation for new mutations. Consistently, our group recently described the novel p.G14R variant associated with an atypical, rapidly progressive phenotype and widespread neuronal loss combined with frontotemporal lobar degeneration–associated aSyn pathology.^24^ To date, limited missense variants have been reported, with some having unresolved pathogenic relevance or requiring independent replication.^21^, ^30^, ^31^ Their identification is further complicated by clinical heterogeneity and incomplete penetrance,^16^, ^32^ resulting in negative family histories. Consequently, validating known variants and identifying novel ones are crucial for improving diagnostic accuracy and genetic counseling. Furthermore, these variants are also pivotal for research elucidating the molecular mechanisms underlying aSyn aggregation and its pathological/physiological properties, including phosphorylation at serine 129 (pS129).^33^, ^34^ For instance, different missense variants enhance or reduce aSyn pathological properties like aggregation and phosphorylation status.^24^, ^35^, ^36^, ^37^ Studying them is key to understanding both physiological and pathological roles of aSyn.

In this study, we identified a novel heterozygous SNCA missense variant in a familial PD case and comprehensively characterize its associated clinical phenotype and molecular effects.

## Patients and Methods

### Clinical Evaluation and Genetic Analysis

The patient was evaluated by movement disorder specialists at the tertiary movement disorder center Paracelsus Elena‐Klinik, Kassel, Germany. Repeated general and neurological examinations included assessment of motor symptoms by Movement Disorder Society–revised Unified Parkinson's Disease Rating Scale (UPDRS), cognition by mini‐mental status test (MMST), and standardized levodopa (l‐dopa) challenge as described previously. Genetic analysis was performed, including whole exome sequencing (WES), to identify causative variants (details are provided in the Supporting Information). Written informed consent was obtained for participation in the genetic study in cooperation with the Institute of Human Genetics, Technical University Munich, Germany. The study was approved by the Ethics Commission of the Landesärztekammer Hessen, Frankfurt am Main, Germany (MC 284/2014). Additional consent was provided for confirmatory genetic testing at the Institute of Human Genetics, University Clinic Göttingen, Germany. Deep brain surgery was performed at the Clinic of Neurosurgery, University Medical Center Göttingen, Germany.

### Biophysical and Cellular Studies

To understand how K58N mutation alters aSyn properties, we combined structural and cellular approaches. Recombinant protein was used for nuclear magnetic resonance (NMR) spectroscopy, Thioflavin T (ThT) aggregation assays, and cryo‐electron microscopy (cryo‐EM) to compare wild‐type (WT) and K58N aSyn structure and fibril formation. Cellular models evaluated effects on aSyn aggregation, condensate formation, membrane association, and S129 phosphorylation. Full methods are provided in the Supporting Information.

## Results

### Clinical Presentation

The index patient, a white man, developed initial motor symptoms at approximately 39 years of age, with left‐sided rest tremor, reduced arm swing, and mild bradykinesia. He was diagnosed with early‐onset PD (EOPD) at the age of 42 years. Dopamine transporter imaging showed an asymmetrical reduction consistent with PD, with no clinical signs of atypical parkinsonism or other neurological disorders. After a good initial response to dopaminergic therapy, the patient developed motor fluctuations with end‐of‐dose wearing off, trunk dyskinesias in the *on* phase, and freezing of gait. Marked dystonia affected the left arm and leg. Thirteen years after PD diagnosis, he was evaluated for deep brain stimulation (DBS). At that time, nonmotor symptoms included excessive daytime sleepiness, fatigue, and depression. Neuropsychological assessment demonstrated no cognitive dysfunction, and a repeated MRI showed no abnormalities or features suggestive of atypical PD. Presurgical l‐dopa challenge test demonstrated a robust response, with 78% improvement of UPDRS Part III. The patient received bilateral DBS of the subthalamic nucleus that reduced motor fluctuations, freezing, and tremor. At 3‐year follow‐up, motor symptom control remained good, with Mini Mental State Examination score 30/30 indicating preserved cognition, although moderate motor fluctuations reemerged. Family history was positive for PD in both a parent and a grandparent, who were deceased but reportedly showed no atypical features or dementia.

### WES Identifies the p.K58N Missense Variant in aSyn

Given the early‐onset of PD and positive family history, the patient underwent WES. WES identified a novel heterozygous missense variant in *SNCA* [NM_000345.4, c.174G>C; p.(K58N)] (Supporting Information Fig. S1A,B). The variant was absent in the genomic databases gnomAD and the PD Variant Browser. It results in a lysine‐to‐asparagine substitution in a highly conserved region (Supporting Information Fig. S1C) and is predicted as deleterious by several in silico prediction algorithms (CADD:31, PolyPhen‐2: 0.994, PrimateAI: 0.8337, REVEL: 0.653).

### The Variant p.K58N Causes a Reduction in α‐Helical Content, Impairing Its Interaction with Liposomes

**FIG. 1:**
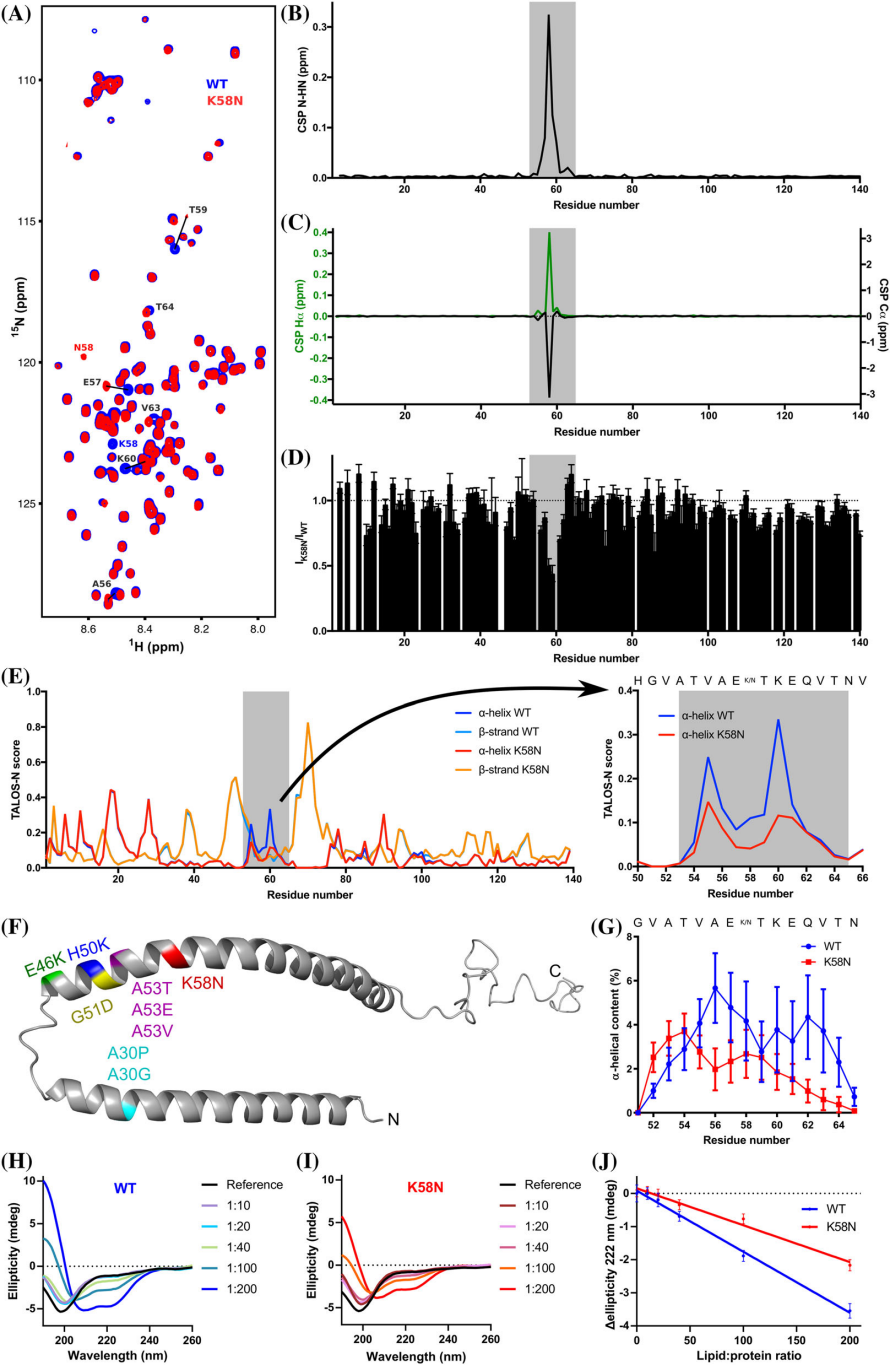
The p.K58N variant decreases the α‐helical content affecting liposome binding. (**A**) ^1^H,^15^N Heteronuclear Single‐Quantum Coherence (HSQC) of α‐synuclein (αSyn) wild type (WT) (blue) and K58N (red). Evident Chemical shift perturbation (CSPs) are labeled. (**B**) Combined HN/N CSPs generated by the K58N mutation over the αSyn sequence. (**C**) Affected region is highlighted in gray. Hα (green) and Cα (black) CSPs. (**D**) Normalized ^1^H,^15^N HSQC intensity ratios. Error bars are calculated from signal‐to‐noise ratios of individual resonances. (**E**) Secondary structure calculations from nuclear magnetic resonance (NMR) chemical shifts for αSyn WT (α‐helix, blue; β‐strand, light blue) and K58N (α‐helix, red; β‐strand, orange). On the right a zoom of the affected region with the α‐helical content is plotted. (**F**) Micelle‐bound αSyn structure (Protein Data Bank [PDB] ID: 1XQ8) with the positions of the different pathological mutations highlighted. (**G**) Residue‐specific α‐helical content over 100‐ns MD simulations. Error bars indicate the standard error from 40 analyzed peptides. (**H–I**) CD experiments of WT (**H**) and K58N (**I**) αSyn at different protein/lipid ratios. (**J**) Ellipticity change at 222 nm on increasing concentrations of lipids for WT (blue) and K58N (red). More negative values indicate a bigger increase of α‐helical structure. [Color figure can be viewed at wileyonlinelibrary.com]

To study the impact of the p.K58N variant on the structural and membrane‐binding properties of aSyn, we employed NMR spectroscopy and Circular dichroism (CD) using recombinantly prepared monomeric WT and K58N aSyn. The two‐dimensional (2D) NMR ^1^H/^15^N‐correlation spectra for both proteins showed minimal signal, consistent with intrinsically disordered proteins (Fig. 1A). Most cross‐peaks overlapped between the two variants, except for those near the mutation site (Fig. 1B–D). Chemical shift perturbations and intensity changes were restricted to the region surrounding the p.K58N substitution (Fig. 1B–D).

To investigate local conformation changes induced by the p.K58N variant, we calculated secondary structure propensity from NMR chemical shifts around the mutation site. Interestingly, the p.K58N variant showed a reduction in the α‐helical content in this region (Fig. 1E). Furthermore, MD simulations of aSyn peptide G51‐N65, either with WT lysine or mutant asparagine at position 58, reproduced NMR data of the full‐length protein, showing decreased helical propensity in the mutant (Fig. 1F). Together, experimental and simulation findings demonstrate that p.K58N locally disrupts the α‐helical structure (Fig. 1E,G).

Because aSyn adopts α‐helical structures upon membrane binding, we assessed the impact of the p.K58N variant on this interaction by incubating aSyn with increasing amounts of liposomes and monitoring secondary structure transitions using CD spectroscopy.^38^, ^39^, ^40^ Liposome exposure resulted in a shift from random coil into α‐helical structure for both variants (Fig. 1H,I). However, the p.K58N variant displayed reduced ellipticity at 222 nm, indicating a lower propensity for α‐helix formation, reflecting reduced liposome binding (Fig. 1J). These findings show α‐helical content is reduced by the variant in a monomeric state, which may explain decreased liposome binding.

### Effect of the Variant p.K58N on aSyn Aggregation In Vitro and Inclusions Formation in Cells

**FIG. 2:**
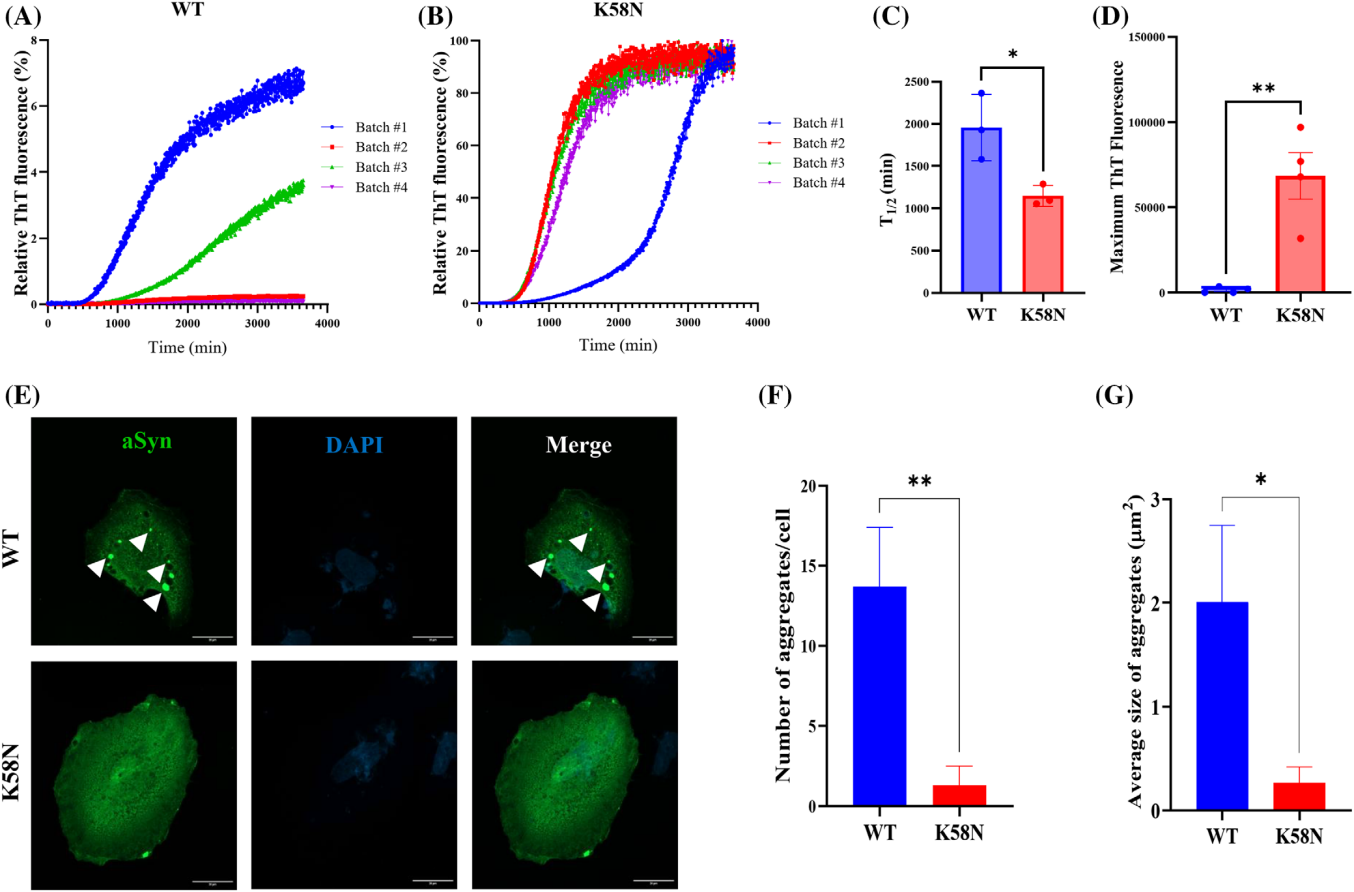
K58N aSyn aggregation propensity. (**A–D**) In vitro ThT‐based aggregation assays for wild type (WT) and K58N α‐synuclein (aSyn). (**A** and **B**) Aggregation kinetic profiles of WT and K58N aSyn. Data were normalized to the maximum fluorescence value of each run. (**C**) Comparison of the half‐time in minutes for aggregation kinetics. (**D**) Comparison of maximum ThT fluorescence values of WT and K58N. Error bars represent mean ± SD. (**E–G**) Effect of K58N mutation on inclusion formation in cells. The patterns of inclusions formation were investigated using the SynT/Sph1 aggregation model, which is based on the coexpression of WT or K58N SynT variants and Sph1. (**E**) Immunohistochemistry images representing the inclusion formation in H4 cells expressing WT and K58N aSyn. Scale bars, 20 μm. (**F**) Quantification of the number of inclusions per cell and their area (**G**). For each experiment, 50 cells were counted at 100× original magnification. The data analysis was performed using a Student's *t* test and presented as mean ± SD (N = 3). *﻿﻿*P* < 0.05, **P < 0.01. [Color figure can be viewed at wileyonlinelibrary.com]

**FIG. 3:**
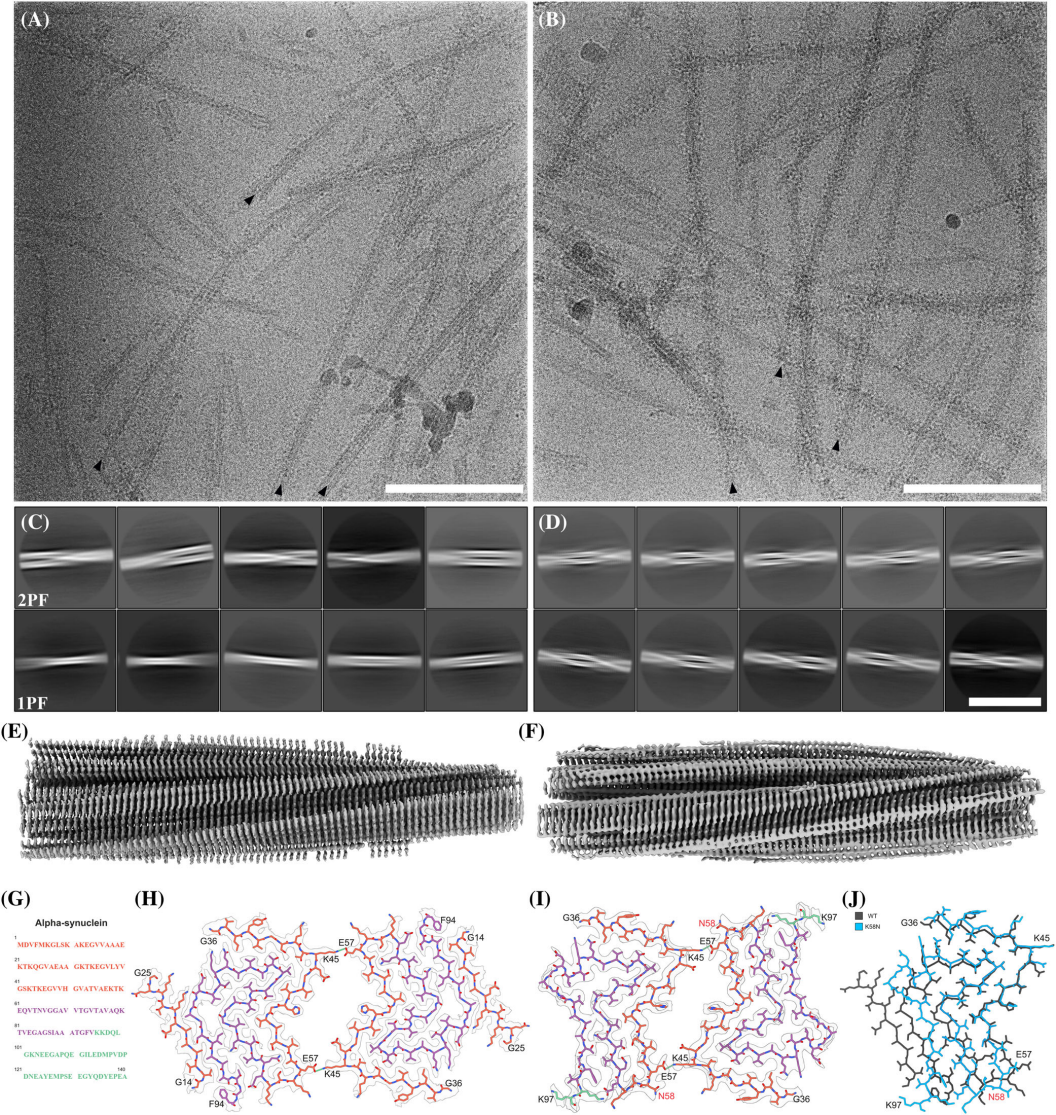
Characteristics of wild‐type (WT) and K58N α‐synuclein (aSyn) filaments. (**A**) Transmission electron microscopy (TEM) micrograph of WT aSyn amyloid filaments. Black arrows mark selected filament ends. Scale bar, 100 nm. (**B**) TEM micrograph of K58N aSyn amyloid filaments. Black arrows indicate filament ends of exemplary filaments. Scale bar, 100 nm. (**C**) Two‐dimensional (2D) class averages (706‐Å box size) of twisting WT aSyn filaments, illustrating the interaction between either two protofilaments (2PF) or a single protofilament (1PF). (**D**) 2D class averages (706‐Å box size) show twisting K58N aSyn filaments, again characterized by two protofilaments interacting with each other. Scale bar, 50 nm. (**E**) Overview of the electron density map of WT aSyn filaments. (**F**) Overview of the electron density map of K58N aSyn filaments. (**G**) Amino acid sequence of human aSyn with distinct regions color‐coded (N terminus in orange, nonamyloid component in purple, and C terminus in green). (**H**) The electron density map together with the atomic model of WT aSyn amyloid filaments featuring a single beta‐sheet layer formed by two interacting protofilaments. The protofilament interface is stabilized by a K45‐E57 salt bridge. (**I**) The electron density map together with the atomic model of K58N aSyn amyloid filaments. The protofilament interface is again stabilized by the K45‐E57 salt bridge. The mutated residue is indicated in red. (**J**) Superposition of the WT atomic model (gray) and the K58N mutant (blue). Although the double‐arrow structure is similar, notable differences in the backbone highlight the impact of the K58N mutation (residue marked in red) on the overall aSyn filament structure. [Color figure can be viewed at wileyonlinelibrary.com]

A central pathological hallmark in PD and other synucleinopathies is the accumulation of aSyn into insoluble aggregates. Therefore, we investigated whether the K58N substitution alters aSyn aggregation propensity. We first applied multiple prediction algorithms, which indicated increased β‐strand content and higher aggregation tendency for K58N (Supporting Information Fig. S2).^41^, ^42^, ^43^ Experimentally, recombinant WT and K58N aSyn were subjected to shaking‐induced fibrillization, monitored by a ThT‐based aggregation assay. Interestingly, the p.K58N variant showed a higher ThT aggregation profile and faster aggregation kinetics with a shorter *t*_₁/₂_ compared with WT (Fig. 2A–D). These findings prompted us to further characterize fibrils by cryo‐EM. As previously reported,^24^ WT aSyn fibrils adopted two similarly populated conformations, one‐ (1PF) and two‐protofilament (2PF) structures, respectively (Fig. 3A,C; Supporting Information Fig. S3). Three‐dimensional (3D) reconstruction was successful only for 2PF fibrils, yielding a structure with 2.7 Å global resolution (Fig. 3E; Supporting Information Figs. S3 and S4) that enabled atomic model building. This model displays a double‐arrow fold previously observed in other studies, such as “polymorph 2A” reported by Guerrero‐Ferreira et al,^44^ stabilized by a salt bridge between lysine 45 (K45) and glutamic acid 57 (E57) at the interprotofilament interface (Fig. 3G,H). The protofilament fold itself is nearly identical to those described as “polymorph L2A/B” by Frieg et al^45^ and “protofilament fold B” by Lövestam et al.^46^ However, in these cases, the interprotofilament interactions differ.

In contrast, the p.K58N variant adopted almost exclusively 2PF conformations (Fig. 3B,D; Supporting Information Fig. S5). A 3D reconstruction at 3.7 Å resolution enabled atomic modeling, demonstrating a protofilament fold similar to that of WT fibrils (Fig. 3F,I,J; Supporting Information Fig. S4). However, K58N fibrils present a higher twist (−1.29 degrees vs −0.81 degree in WT) and a slightly shifted protofilament interface, possibly because of the proximity of the K58N substitution to the interface‐stabilizing residue (E57). In addition, no density for residues 14 to 25 was visible at the periphery of mutant fibrils (Fig. 3G–J). Altogether, these data indicate that the p.K58N variant displaces the conformational equilibrium of aSyn fibrils toward a 2PF structure reminiscent of that observed for WT.

Following the in vitro aggregation analysis, we explored whether the p.K58N variant exhibits altered aggregation behavior under a cellular environment, using the established SynT/Synphilin‐1 (Sph1) model, a widely used cellular system for studying aSyn inclusions formation.^47^ Human neuroglioma cells (H4) were cotransfected with WT or K58N SynT constructs and Sph1, followed by immunostaining 48 hours posttransfection. Interestingly, the K58N variant exhibited a significant decrease in the number and size of aSyn inclusions compared with WT (Fig. 2E–G) and an increased percentage of cells without inclusions compared with WT (Supporting Information Fig. S6).

### K58N aSyn Shows Decreased Phase Separation

**FIG. 4:**
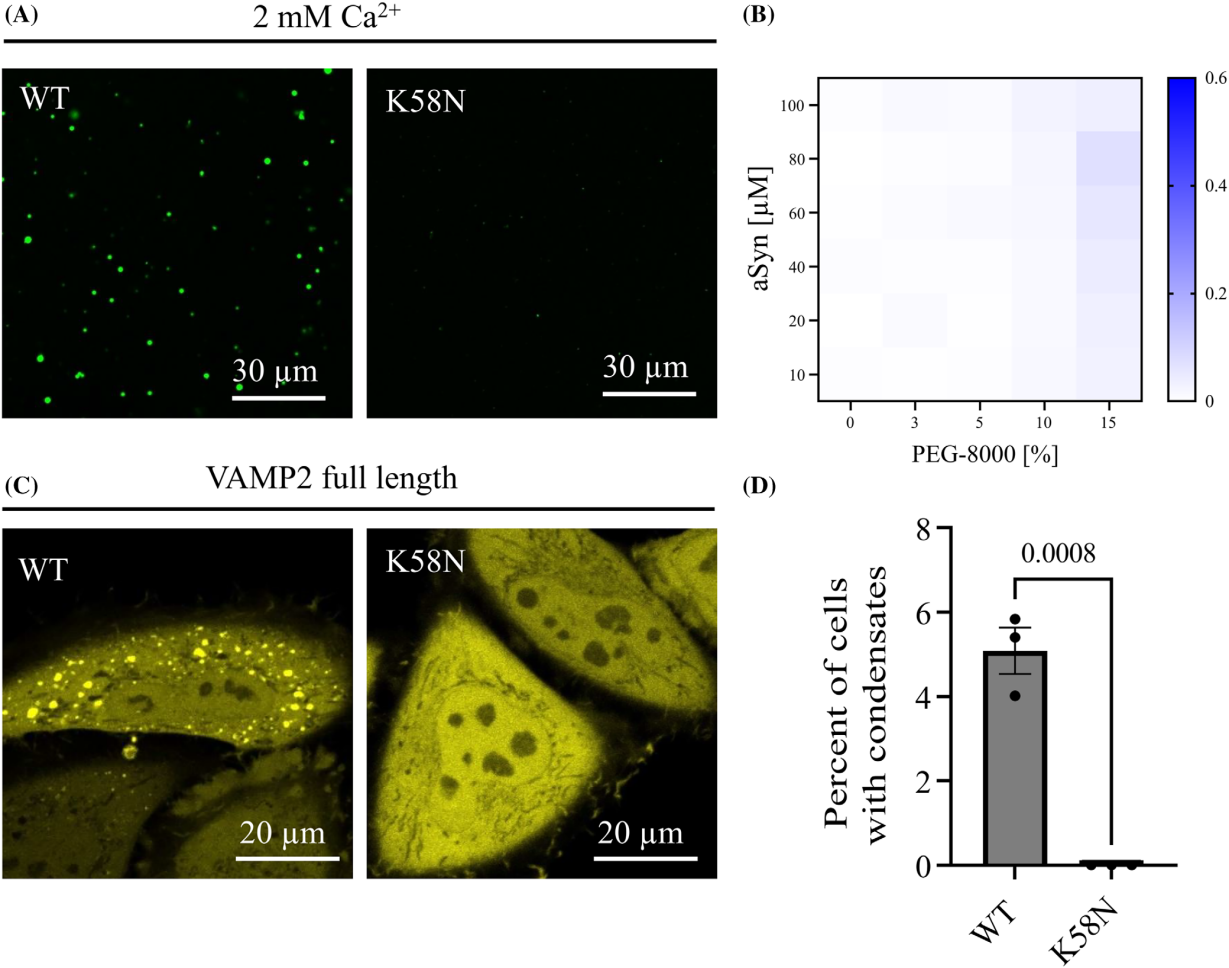
aSyn K58N shows decreased condensate formation in vitro and in cells. (**A**) aSyn phase separation in the presence of 2 mM Ca^2+^ and crowding with 15% PEG‐8000, immediately after PEG addition for aSyn wild‐type (WT) and the disease variant aSyn K58N. aSyn concentration used: 100 μM. (**B**) Heatmap for turbidity measurements of aSyn phase separation in the presence of 2 mM Ca^2+^. Data are derived from four independent repeats. (**C**) Condensate formation of aSyn WT YFP and aSyn K58N YFP on ectopic expression with VAMP2 in HeLa cells. aSyn K58N YFP does not undergo condensate formation in cells. (**D**) Quantification of condensate formation. Data were derived from incuCyte screening, 16 images per well, three wells per biological repeat, three biological repeats. n indicates biological repeats. Data are represented as mean ± SD. Unpaired two‐tailed *t* test. [Color figure can be viewed at wileyonlinelibrary.com]

We further explored the impact of this novel variant on aSyn phase separation, a critical factor that is thought to play a role in its functional and pathological behavior. Under conditions promoting droplet formation, K58N aSyn exhibits markedly reduced droplet formation compared with WT aSyn (Fig. 4A). This is recapitulated at a quantitative scale, by turbidity assays in the presence of Ca^2+^ and varying Polyethylene glycol (PEG) concentrations (Fig. 4B).

We next expressed aSyn YFP with VAMP2 in HeLa cells, where VAMP2 induces aSyn phase separation as previously shown.^48^ In this study, WT aSyn YFP formed condensates, whereas K58N aSyn YFP failed to form condensates exhibiting a homogenous cytosolic distribution (Fig. 4C). Quantitative evaluation confirms the absence of condensate formation for the K58N aSyn variant (Fig. 4D).

### Effects of the Variant p.K58N on aSyn pS129

**FIG. 5:**
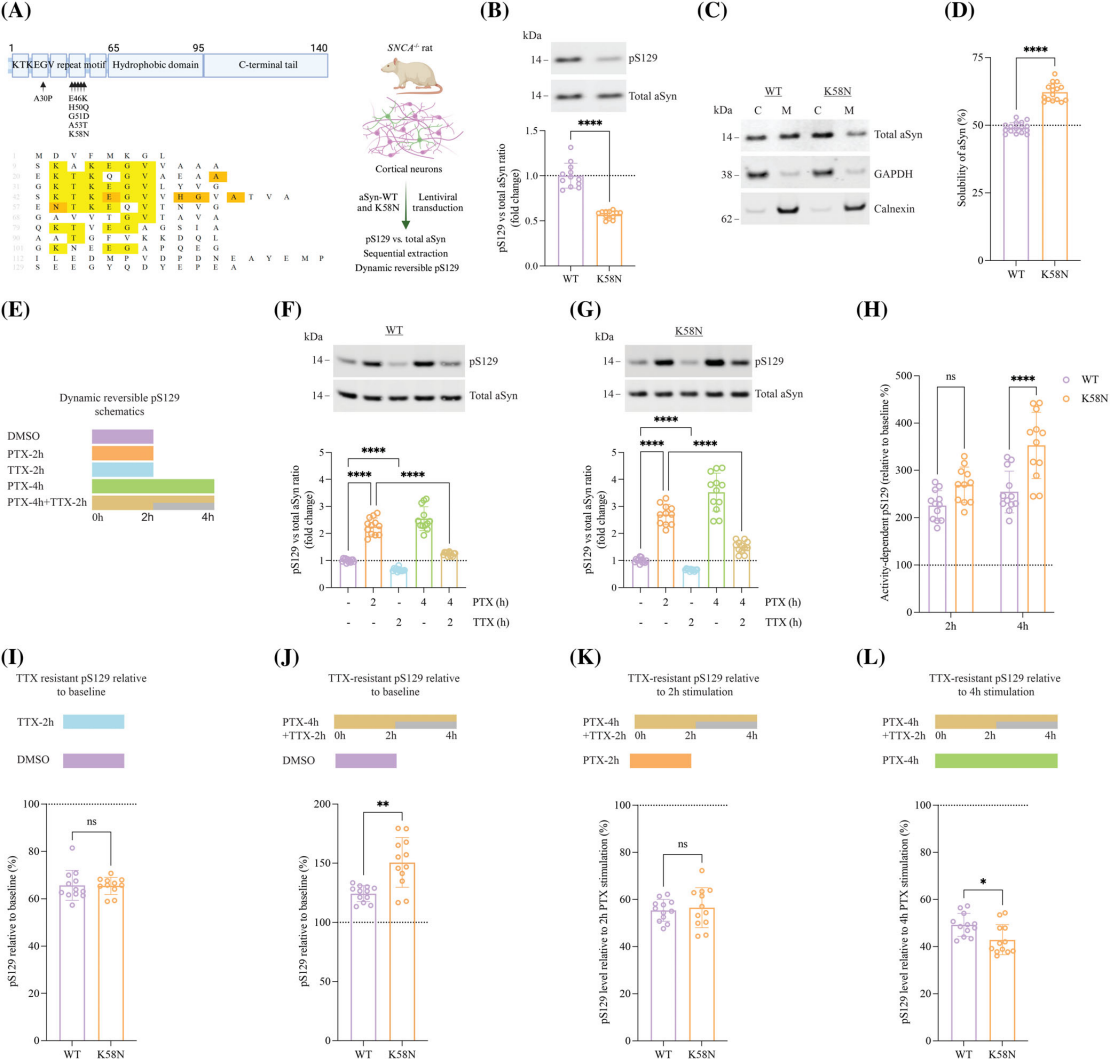
Dynamic activity‐dependent phosphorylation at serine 129 (pS129) of K58N and wild‐type (WT) α‐synuclein (aSyn). (**A**) Schematic illustration of aSyn structure exhibiting the KTKEGV repeat sequence harboring most familial PD mutations, central hydrophobic domain, and C‐terminal region. The sequence alignment of aSyn is displayed below showing the conserved KTKEGV residues in yellow and sites of familial PD mutation in orange. On the right, the experimental setup is summarized. (**B**) A representative Western blot (WB) displaying the levels of total aSyn and pS129 from DIV 17–21 rat *SNCA* ^−/−^ cortical neurons expressing WT and K58N aSyn, with WB quantitative analysis presented below. (**C**) WB results of WT and K58N transduced rat *SNCA* ^−/−^ cortical neurons that were subjected to on‐plate sequential extraction to isolate the cytosolic (C) and membrane (M) fractions. MJFR1 antibody was used for total aSyn detection, whereas GAPDH and Calnexin were used as cytosolic and membrane fractions controls, respectively. (**D**) Quantitative analysis of WT and K58N aSyn solubility from WB data in (**C**). (**E**) A summary of the experimental conditions to study pS129 dynamic reversibility, with more information provided in the main text. (**F** and **G**) Neuronal activity‐dependent reversible phosphorylation of S129 (as outlined in **E**) was detected in DIV 17–21 rat *SNCA* ^*−/−*^ cortical neurons expressing WT or K58N, respectively, after picrotoxin (PTX) stimulation (20 μM) and tetrodotoxin (TTX) inhibition (1 μM). WB for quantifying total aSyn and pS129 was employed, and (**H**)–(**L**) are derived from (**F**) and (**G**). (**H**) The percentage of increase in pS129, compared with baseline, for WT and K58N aSyn observed after 2 or 4 h of PTX stimulation. (**I**) Comparison of TTX‐resistant pS129 levels in WT and K58N variants relative to baseline conditions (DMSO vehicle). (**J**) The proportion of irreversible pS129 relative to the basal (DMSO vehicle) condition. (**K**) The proportion of irreversible pS129 relative to 2 h of PTX stimulation. (**L**) The proportion of irreversible pS129 relative to 4 h of PTX stimulation. ****P < 0.0001, ***P < 0.001, *P < 0.05. The error bar was mean ± SD. ns, not significant. [Color figure can be viewed at wileyonlinelibrary.com]

There is increasing evidence that pS129 of aSyn is not only associated with pathology but also influences the physiological function of aSyn.^34^ To investigate whether the p.K58N variant alters pS129 status, we employed lentiviral vectors to express either human WT or K58N aSyn in primary aSyn knockout (*SNCA* ^−/−^) rat cortical cultures (Fig. 5A). Under basal conditions (ie, without neuronal stimulation), K58N aSyn exhibited significantly reduced pS129 levels compared with WT (Fig. 5B). Recent studies showed familial PD‐associated aSyn mutants with reduced membrane (M) localization often exhibit lower basal pS129 levels.^34^ In line with this, we observed that the variant p.K58N was enriched in the cytosolic fraction (C) (GAPDH fraction, ~ 62%) (Fig. 5C,D), supporting the idea that reduced membrane association contributes to lower pS129 levels.

pS129 also has been recently reported to a dynamic, activity‐dependent physiological process, where pS129 increases after neuronal stimulation and reversibly returns to baseline once the stimulus dampens or is inhibited.^34^ Importantly, rodent neuron cultures expressing PD‐associated A30P and E46K aSyn mutants showed impaired dynamic reversibility of pS129.^49^ Therefore, we assessed the effect of K58N on activity‐dependent pS129 dynamics by exposing WT or K58N aSyn transduced rat *SNCA* ^−/−^ cortical neurons at days in vitro (DIV) 17 to 21 to neuronal stimulation with picrotoxin (PTX; a GABA_A receptor antagonist), inhibition using tetrodotoxin (TTX; a sodium channel blocker), or a combination of stimulation followed by inhibition (Fig. 5E). Consistent with our previous observations, PTX exposure for 2 or 4 hours significantly increased pS129 in WT neurons, whereas TTX decreased basal pS129 by ~30%. TTX inhibition 2 hours after PTX stimulation effectively reversed activity‐dependent pS129 elevation (Fig. 5F). K58N neurons followed a similar activity‐dependent pS129 response (Fig. 5G); however, 4‐hour PTX treatment induced a more pronounced pS129 increase in K58N neurons than in WT, relative to baseline (Fig. 5H). To assess pS129 dynamic reversibility, we compared pS129 levels after 4‐hour PTX with midpoint TTX inhibition with those after 2‐ or 4‐hour PTX alone. The percentage of irreversible pS129 after PTX/TTX was similar between K58N and WT neurons at 2 hours (Fig. 5K), but lower in K58N neurons after 4 hours (Fig. 5L). TTX inhibition under unstimulated conditions showed no significant difference between K58N and WT neurons (Fig. 5I). Overall, our findings indicate that activity‐dependent pS129 dynamics are altered in K58N neurons, particularly after prolonged neuronal activity.

### aSyn Containing the Variant p.K58N Is More Cytosolic without Changing Cytotoxicity in Yeast Cells

To investigate how the K58N aSyn variant affects cellular distribution and cytotoxicity, we used the well‐established budding yeast model to associate subcellular localization with toxicity.^50^ K58N aSyn predominantly localized to the cytoplasm, whereas WT aSyn formed cytoplasmic inclusions (Supporting Information Fig. S7A). Despite the different subcellular localization, the K58N and WT aSyn presented identical toxicities (Supporting Information Fig. S7).

## Discussion

We report a novel *SNCA* missense variant (c.174G>C; p.K58N) and provide a detailed description of its clinical phenotype and molecular effects. The variant was identified through WES in a patient with EOPD and a positive family history, suggesting a genetic cause. Although both affected relatives were already deceased, DNA was unavailable for segregation analysis; the pathogenicity of the K58N is supported by its absence in genomic databases, disruption of a highly conserved residue, pathogenic predictions from multiple in silico tools, and critically, our molecular characterization results. According to ACMG (American College of Medical Genetics) guidelines,^51^ p.K58N is classified as a variant of unknown significance. Our data now clarify its molecular consequences.

The clinical presentation and progression resembled idiopathic PD, with a good and sustained l‐dopa response and the lack of motor and nonmotor symptoms indicating atypical PD. Both the parent and the grandparent were also diagnosed with PD; although both were already deceased and could not be examined, their medical history, as reported by the index patient, was consistent with idiopathic PD. Known pathogenic *SNCA* missense variants exhibit phenotypic spectrum ranging from typical idiopathic PD to atypical and more complex syndromes.^29^ Atypical symptoms are common in patients with some variants such as p.G51D,^18^, ^52^ but rarely described in p.A30P and p.A30G,^22^, ^53^ which are associated with a more benign disease course compared with other missense variants, similar to our identified variant. However, unlike those variants, which often show early cognitive decline, no such symptoms were observed in this study. Motor fluctuations, common across all *SNCA* mutations including multiplications and observed in about two‐thirds of cases,^26^ were also present in the p.K58N patient and ultimately required the implantation of bilateral nucleus subthalamicus DBS. The intervention provided a good overall benefit for motor symptoms and motor fluctuations. Our study demonstrates DBS benefit in a p.K58N carrier, but recurrence of motor fluctuations after 4 years underscores the need for long‐term follow‐up. Emerging evidence indicates DBS outcomes may be affected by specific genetic mutations,^54^ yet data on *SNCA* missense variants remain limited to a few cases and are inconsistent.^22^, ^55^, ^56^, ^57^, ^58^ Although short‐term motor improvement is consistently reported for DBS, two unrelated p.A53E patients experienced post‐DBS progression for motor and nonmotor symptoms.^55^, ^57^ Reemergence of motor fluctuations after DBS was also reported in two patients with p.A30G variant (after 3 years)^22^ and p.E46K (unknown interval),^58^ but both had less aggressive progression than p.A53E cases. Additional data are needed to clarify how *SNCA* variants affect DBS outcomes, which is important for patient counseling and indication for DBS.

Most PD‐associated aSyn variants occur in the N‐terminal domain, a region crucial for interaction with lipids and biological membranes. Binding to membranes is mediated by KTKEGV repeats, which form amphipathic α‐helices essential for lipid binding and key to aSyn structural and functional dynamics in cells. Under normal conditions, aSyn transitions between a cytosolic soluble disordered form and membrane‐bound helical conformations.^40^, ^59^ N‐terminal mutations often disrupt this balance by altering helical propensity, affecting membrane binding versus cytosolic solubility, and thus aSyn function and pathology.^47^, ^60^ The p.K58N variant is located in the N‐terminal region, specifically within the KTKEGV motif, where lysine (K), a positively charged residue important for α‐helix formation through electrostatics interactions with lipid headgroups in the membranes, is replaced by uncharged asparagine (N). This substitution is expected to disrupt α‐helix structure and membrane binding.^61^ Asparagine also favors turns and coils, potentially further destabilizing local structure. Consistently, our NMR data showed K58N mutation locally reduced α‐helical content in residues A53–N65. Simulation data complemented the NMR findings, supporting disruption of aSyn helical propensity near the mutation site. The K58N‐induced changes in conformational dynamics are expected to impact aSyn functional properties including its membrane interactions. However, the impact of PD‐associated mutations on membrane binding varies and is further influenced by other factors such as membrane lipid composition and curvature. Among PD mutations, A30P is known to exhibit defective membrane binding by reducing helical propensity.^62^, ^63^ In this study, K58N reduced α‐helical propensity in the presence of liposomes compared with WT aSyn, indicating a lower‐affinity lipid‐binding affinity.

A central hallmark of synucleinopathies is misfolding and aggregation of aSyn into insoluble, fibrillar protein deposits. Furthermore, distinct fibrils strains were reported across different synucleinopathies, prompting us to investigate p.K58N variant impact on aSyn assembly. Importantly, this variant affects a residue adjacent to the central hydrophobic domain, a key region for aSyn aggregation. Furthermore, K58N local effects extended beyond the mutation site to residues farther away, including those at the start of the hydrophobic domain. Aggregation prediction algorithms suggest that K58N exhibits higher aggregation propensity, consistent with our in vitro aggregation assays showing increased ThT fluorescence and altered aggregation kinetics compared with WT aSyn. Substitution of positively charged K at position 58 with uncharged asparagine may remove protection against aggregation. This is reminiscent of the H50Q variant, where replacing partially charged histidine with neutral glutamine similarly increased aggregation tendency in vitro.^64^, ^65^

The observed changes likely reflect distinct structural properties of fibrils formed by WT and K58N aSyn. Cryo‐EM analysis demonstrated that p.K58N preferentially forms 2PF fibrils with a similar protofilament fold than WT fibrils, suggesting that the mutation stabilizes the WT interprotofilament interface. One possibility is that K58 modulates the ability of the adjacent interface‐stabilizing residue (E57) to interact in *cis* or *trans*. In WT, K58 may form a salt bridge in *cis* with E57, potentially destabilizing the interprotofilament interface and thereby favoring 1PF structures. In contrast, the p.K58N variant may prevent this interaction because of the altered charge properties, thereby promoting *trans* interactions with K45 of an adjacent protofilament and thus favoring 2PF fibrils. Interestingly, this contrasts with our recent findings on the G14R mutation,^24^ which promotes 1PF forms. Altogether, these results indicate that these mutations modulate the WT fibril conformational landscape, favoring either 1PF (G14R) or 2PF (K58N) forms.

In cells, K58N aSyn formed fewer and smaller inclusions compared with WT aSyn. This aligns with previous findings where some aSyn mutations showed attenuated aSyn aggregation in vitro but increased cellular inclusions.^24^, ^47^ The yeast model also showed a more diffuse cytoplasmic distribution for K58N, further demonstrating the importance of employing diverse experimental models when evaluating the effects of mutations in the behavior of aSyn, because this will likely uncover different properties of the protein.

aSyn phase separation forms dynamic condensates under physiological conditions,^66^, ^67^ but in certain pathological states, it can result in the formation of precursors or seeds that develop into solid, insoluble pathological aggregates.^68^, ^69^, ^70^ Recent studies show that VAMP2 regulates aSyn phase separation condensate formation, likely through aSyn membrane interactions.^48^, ^71^ In these studies, A30P mutant, known for reduced lipid binding^61^, ^72^, ^73^, ^74^ and increased cytosolic localization,^50^, ^75^ failed to form condensates in cells. Interestingly, our assays showed that the K58N mutant also has impaired membrane binding and increased cytosolic distribution, similar to A30P.

The phosphorylation of aSyn at S129 (pS129), previously recognized as a pathological hallmark,^33^ is increasingly recognized for its physiological role.^34^, ^76^ This phosphorylation is a dynamic reversible process regulated by neuronal activity, increasing with stimulation, and reversing when activity diminishes.^34^ In our study, both WT and K58N aSyn exhibited similar activity‐dependent pS129 patterns, aligning with previous findings for some PD‐associated mutations.^24^, ^49^ These findings support mutation‐specific pathological effects and align with the more benign disease course observed in the patient. These results underscore the importance of evaluating pS129 dynamics to better understand the molecular impact of aSyn mutations.

The identification and characterization of novel aSyn variants are very important for the field of synucleinopathies, because this not only sheds new light onto our understanding of pathological mechanisms, but it also enables the clinical stratification of patients (for clinical trials). In addition, this knowledge of disease‐associated aSyn variants is important for genetic counseling, once the pathogenicity is confirmed, and may guide the application of potential future individualized therapies.

## Author Roles

(1) Research Project: A. Conception, B. Organization, C. Execution; (2) Statistical Analysis: A. Design, B. Execution, C. Review and Critique; (3) Manuscript Preparation: A. Writing of the First Draft, B. Review and Critique.

M.A.: 1C, 2A, 2B, 2C, 3A, 3B.

S.W.: 1C, 2A, 2B, 3A, 3B.

N.R.: 1C, 2A, 2B, 2C, 3B.

M.R.: 1C, 3B.

L.S.: 1C, 3B.

G.M.: 1C, 3B.

M.Z.: 1C, 3B.

K.S.: 1C, 2B, 3B.

A.I.O.: 1C, 2B, 3B.

V.J.: 1C, 3B.

L.A.: 1C, 3B.

A.A.: 1C, 3B.

A.C.: 1C, 3B.

S.R.C.: 2B, 2C, 3B.

J.W.: 2C, 3B.

C.T.: 2C, 3B.

M.S.: 1C, 3B.

S.P.: 2C, 3B.

U.D.: 2A, 2C, 3B.

C.O.F.: 2C, 3B.

J.L.: 2A, 2C, 3B.

M.Z.: 2A, 2C, 3B.

R.F.B.: 2A, 2C, 3B.

B.M.: 1A, 1B, 2A, 2C, 3B.

T.F.O.: 1A, 1B, 2A, 2C, 3A, 3B.

## Financial Disclosures of All Authors

The authors report no financial disclosures or conflicts of interest for the past 12 months.

## Supporting information


**Fig S1. Identification of K58N mutation**. (A) and (B) present DNA sequencing results of the SNCA gene, Exon 4, from a wildtype control sample (A) and a patient sample (B) with a heterozygous variant c.174G>C; S = G or C. (C) illustrates conservation of the SNCA K58N missense mutation across various species.

**Fig S2. Prediction of the impact of the K58N mutation on structural and aggregation properties of aSyn**. (A) Evaluation of the effect of K58N using PASTA 2.0 algorithm. The mutation tends to reduce the amount of α‐helix and number coils, and to increase the β‐strand content. (B) Prediction of amyloidogenic sequences of WT (left) and K58N (right) aSyn through FoldAmyloid tool based on the probability of formation of hydrogen bonds. A slight increase in the aggregation probability was observed for K58N variant. (C) Analysis of aggregation‐prone regions for WT and K58N aSyn (D) utilizing GAP (Aggregation Proneness) prediction algorithm, which is based on analyzing each part of the protein as a 6‐amino acid long peptide and indicating which is the probability of the peptide forming an amyloid structure. According to GAP, there is an increase in the individual probability of each peptide to form amyloid structures, with exception to the peptide in the position 56, for K58N compared to WT aSyn.

**Fig S3. Detailed Processing Workflow for the WT aSyn Dataset**. (A) From the initially picked helical segments, a subset was excluded due to artifacts, such as the presence of carbon edges. The remaining segments were sorted into two categories based on structural features: segments exhibiting two protofilaments (2PF, “wide”) and those with a single protofilament (1PF, “narrow”). (B) During the initial classification step, which utilized thrice‐binned segments, the distribution of segments between the two groups was approximately equal, as depicted in the associated pie chart. (C) Further classification performed on unbinned data confirmed the results of the initial classification, with segments evenly divided between the 2PF and 1PF categories, as illustrated in the corresponding pie chart. (D) The three‐dimensional classification results are shown, including the final electron density map of the 2PF data following 3D refinement, postprocessing, and CTF refinement. The accompanying pie chart reveals a slightly greater contribution of segments to the 2PF (two‐protofilament) structure compared to the 1PF structure.

**Fig S4.** Fourier Shell Correlation (FSC) curves for 2PF WT (A) and 2PF K58N (B).

**Fig S5.** Detailed Processing Workflow for the K58N aSyn Dataset. (A) A subset of the initially picked helical segments was removed due to artifacts, such as those containing carbon edges. The remaining segments were divided into two categories based on their structural features: segments showing two protofilaments (2PF, “wide”) and those showing a single protofilament (1PF, “narrow”). (B) During the initial classification step with three times binned segments, a significant majority of the segments were categorized as 2PF, with only a small fraction classified as 1PF, as illustrated by the provided pie chart. (C) Subsequent classification with unbinned data supported the initial results, as depicted by the adjacent pie chart. In contrast to the 2PF dataset, no clear beta‐sheet separation was observed in the unbinned class averages for the 1PF dataset, hinting at the lower quality and quantity of the 1PF segments. (D) The results of the three‐dimensional classification are presented. For the 2PF data, the refined electron density map is shown after 3D refinement, postprocessing, and CTF refinement. Due to the poor quality of particles and class averages in the 1PF dataset, further processing via 3D classification was not feasible.

**Fig S6. Effect of K58N mutation on inclusion formation in cells. (A)** Representative immunohistochemistry images of H4 cells showing the patterns of inclusion formation for both WT and K58N SynT variants. Scale bar: 20 μm. (B) and (C) show the quantification of the number of inclusions from transfected cells. A total of 50 cells were counted using a 20x objective for each experiment, and classified into four groups based on their inclusion patterns. Student's t‐test was used to analyze the data, and the results are shown as mean ± SD from 3 independent experiments.

**Fig S7. Effect of K58N mutation on yeast cells**. S. cerevisiae cells harboring WT aSyn or K58N aSyn mutation were grown to mid‐log phase. (A) aSyn localization was analyzed by fluorescence microscopy. (B) Cellular viability was evaluated by spotting assay, where cultures were spotted on SD‐URA agar plates.

Supplementary Information.

## Data Availability

The main data generated or analyzed during this study are included in this article (and its Supporting Information files). The micrographs used for the single‐particle analysis (SPA) of aSyn fibrils are available in the EMPIAR database under accession code EMPIAR‐12518. The atomic model and cryo‐EM density map for the WT aSyn fibril are deposited in the Protein Data Bank (PDB) and Electron Microscopy Data Bank (EMDB) under accession codes 9HGS and EMD‐52165, respectively. The corresponding data for the K58N mutant aSyn fibril are available under accession codes 9HXA (PDB) and EMD‐52458 (EMDB). A comprehensive Key Resource Table, detailing datasets, software, and protocols, is available via Zenodo at: https://zenodo.org/records/14730688. In addition, the entry includes an Excel file with tabular data on the protofilament distribution of WT and K58N aSyn filaments, XML files containing tabular data for the FSC graphs of each density map used to determine the final resolution (generated with RELION 4.0), and two Python scripts for graphing the FSC XML data. The full single‐particle analysis protocol describing the cryo‐EM data processing strategy is available at Protocols.io (https://www.protocols.io/view/single-particle-analysis-of-synuclein-fibrils-81wgbxozylpk/v1).
