# Supplementary material for: A Novel α‐Synuclein K58N Missense Variant in a Patient with Parkinson's Disease
Source: Mov Disord. 2025 Sep 4;40(12):2732–45. doi: 10.1002/mds.70030 (PMC12710137; doi:10.1002/mds.70030)
Supplement: Supplementary file 1 — Fig S1. Identification of K58N mutation. (A) and (B) present DNA sequencing results of the SNCA gene, Exon 4, from a wildtype control sample (A) and a patient sample (B) with a heterozygous variant c.174G>C; S = G or C. (C) illustrates conservation of the SNCA K58N missense mutation across various species. [file MDS-40-2732-s001.pdf]

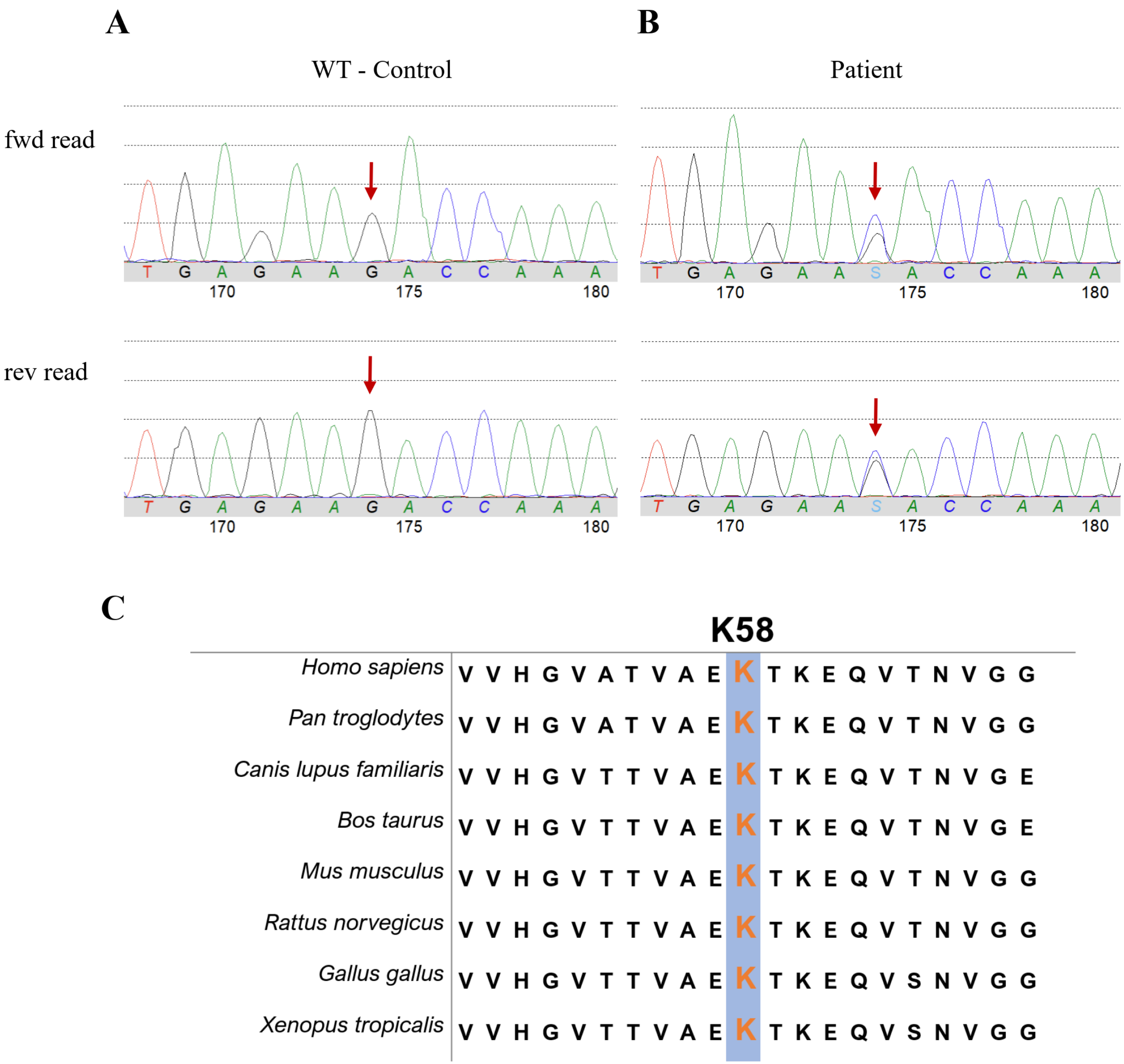
A
B
WT - Control
Patient
fwd read
rev read
T G A G A A G A C C A A A
170 175 180
T G A G A A S A C C A A A
170 175 180
T G A G A A G A C C A A A
170 175 180
T G A G A A S A C C A A A
170 175 180
C
K58
Homo sapiens V V H G V A T V A E K T K E Q V T N V G G
Pan troglodytes V V H G V A T V A E K T K E Q V T N V G G
Canis lupus familiaris V V H G V T T V A E K T K E Q V T N V G E
Bos taurus V V H G V T T V A E K T K E Q V T N V G E
Mus musculus V V H G V T T V A E K T K E Q V T N V G G
Rattus norvegicus V V H G V T T V A E K T K E Q V T N V G G
Gallus gallus V V H G V T T V A E K T K E Q V S N V G G
Xenopus tropicalis V V H G V T T V A E K T K E Q V S N V G G
