# Supplementary material for: A Novel α‐Synuclein K58N Missense Variant in a Patient with Parkinson's Disease
Source: Mov Disord. 2025 Sep 4;40(12):2732–45. doi: 10.1002/mds.70030 (PMC12710137; doi:10.1002/mds.70030)
Supplement: Supplementary file 2 — Fig S2. Prediction of the impact of the K58N mutation on structural and aggregation properties of aSyn. (A) Evaluation of the effect of K58N using PASTA 2.0 algorithm. The mutation tends to reduce the amount of α‐helix and number coils, and to increase the β‐strand content. (B) Prediction of amyloidogenic sequences of WT (left) and K58N (right) aSyn through FoldAmyloid tool based on the probability of formation of hydrogen bonds. A slight increase in the aggregation probability was observed for K58N variant. (C) Analysis of aggregation‐prone regions for WT and K58N aSyn (D) utilizing GAP (Aggregation Proneness) prediction algorithm, which is based on analyzing each part of the protein as a 6‐amino acid long peptide and indicating which is the probability of the peptide forming an amyloid structure. According to GAP, there is an increase in the individual probability of each peptide to form amyloid structures, with exception to the peptide in the position 56, for K58N compared to WT aSyn. [file MDS-40-2732-s002.pdf]

A

Parameters	WT aSyn	K58N aSyn
Length	140	140
#Amyloids	20	20
Best energy	-7.239327	-7.239327
% disorder	34.28	34.28
% α-helix	26.43	20.71
% β-strand	22.14	25.71
%coil	51.43	53.57

B

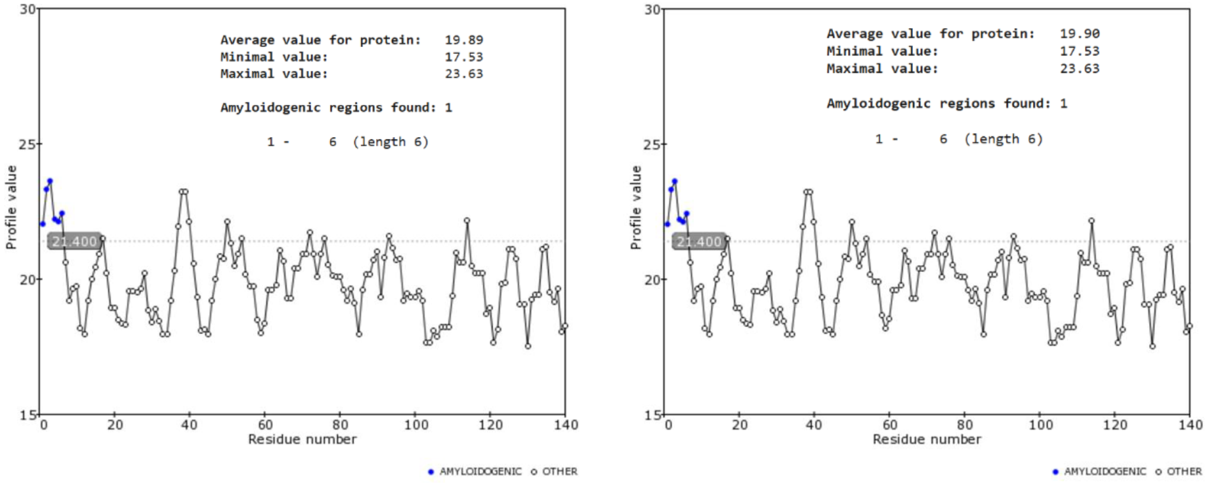

C

No.	Peptide	Amyloid probability	Non-amyloid probability
53	ATVAEK	0.740	0.260
54	TVAEKT	0.172	0.828
55	VAEKTK	0.971	0.029
56	AEKTKE	0.823	0.177
57	EKTKEQ	0.989	0.011
58	KTKEQV	0.999	0.001

D

No.	Peptide	Amyloid probability	Non-amyloid probability
53	ATVAEN	0.992	0.008
54	TVAENT	0.771	0.229
55	VAENTK	0.954	0.046
56	AENTKE	0.024	0.976
57	ENTKEQ	0.999	0.001
58	NTKEQV	1.000	0.000
