# Supplementary material for: A Novel α‐Synuclein K58N Missense Variant in a Patient with Parkinson's Disease
Source: Mov Disord. 2025 Sep 4;40(12):2732–45. doi: 10.1002/mds.70030 (PMC12710137; doi:10.1002/mds.70030)

Supplementary Figure - Processing Overview WT

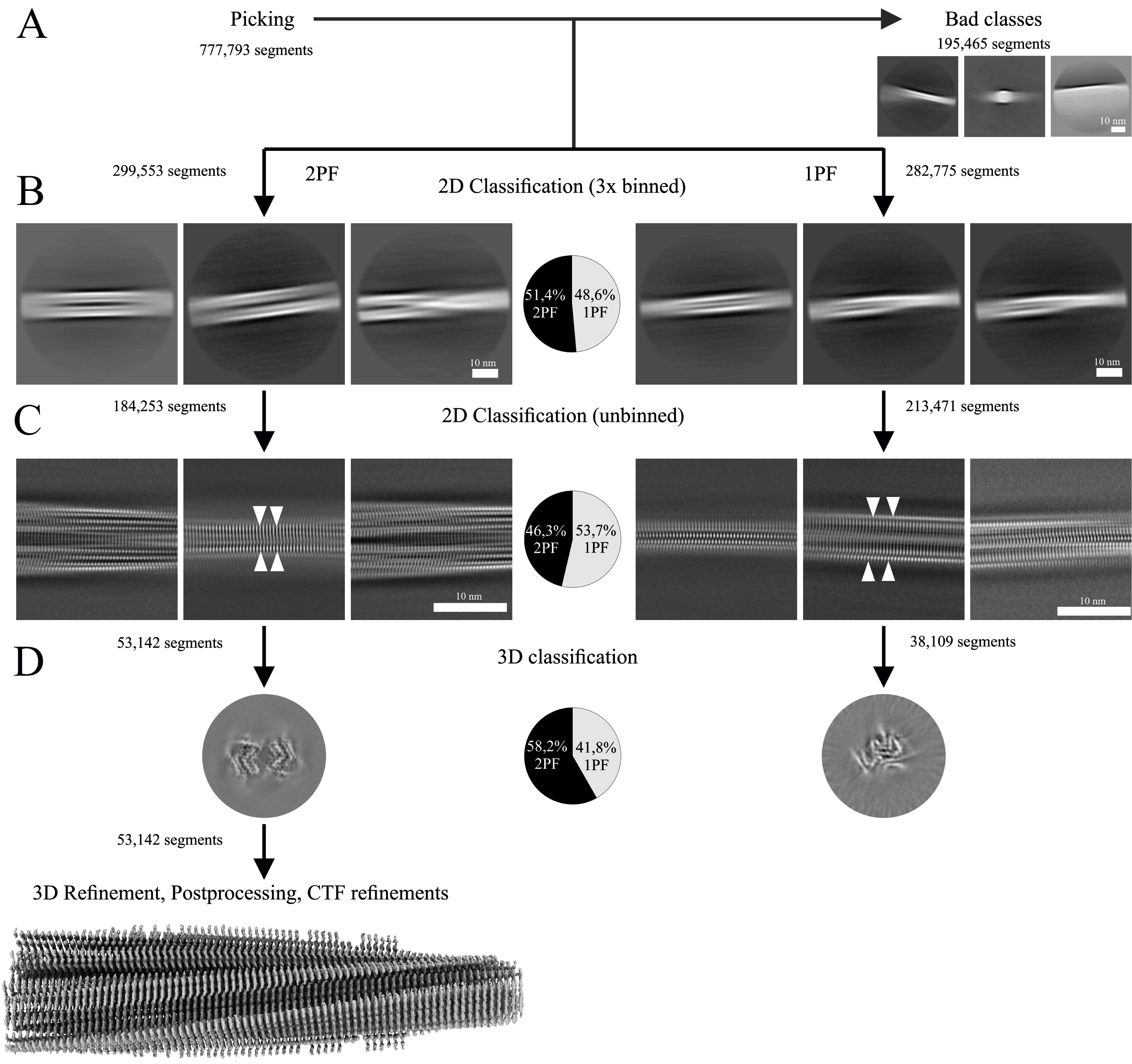

Supplement: Supplementary file 3 — Fig S3. Detailed Processing Workflow for the WT aSyn Dataset. (A) From the initially picked helical segments, a subset was excluded due to artifacts, such as the presence of carbon edges. The remaining segments were sorted into two categories based on structural features: segments exhibiting two protofilaments (2PF, “wide”) and those with a single protofilament (1PF, “narrow”). (B) During the initial classification step, which utilized thrice‐binned segments, the distribution of segments between the two groups was approximately equal, as depicted in the associated pie chart. (C) Further classification performed on unbinned data confirmed the results of the initial classification, with segments evenly divided between the 2PF and 1PF categories, as illustrated in the corresponding pie chart. (D) The three‐dimensional classification results are shown, including the final electron density map of the 2PF data following 3D refinement, postprocessing, and CTF refinement. The accompanying pie chart reveals a slightly greater contribution of segments to the 2PF (two‐protofilament) structure compared to the 1PF structure. [file MDS-40-2732-s003.pdf]
