# Supplementary figures and images for: A Novel α‐Synuclein K58N Missense Variant in a Patient with Parkinson's Disease
Source: Mov Disord. 2025 Sep 4;40(12):2732–45. doi: 10.1002/mds.70030 (PMC12710137; doi:10.1002/mds.70030)

**A**

Final resolution = 2.7 Angstroms

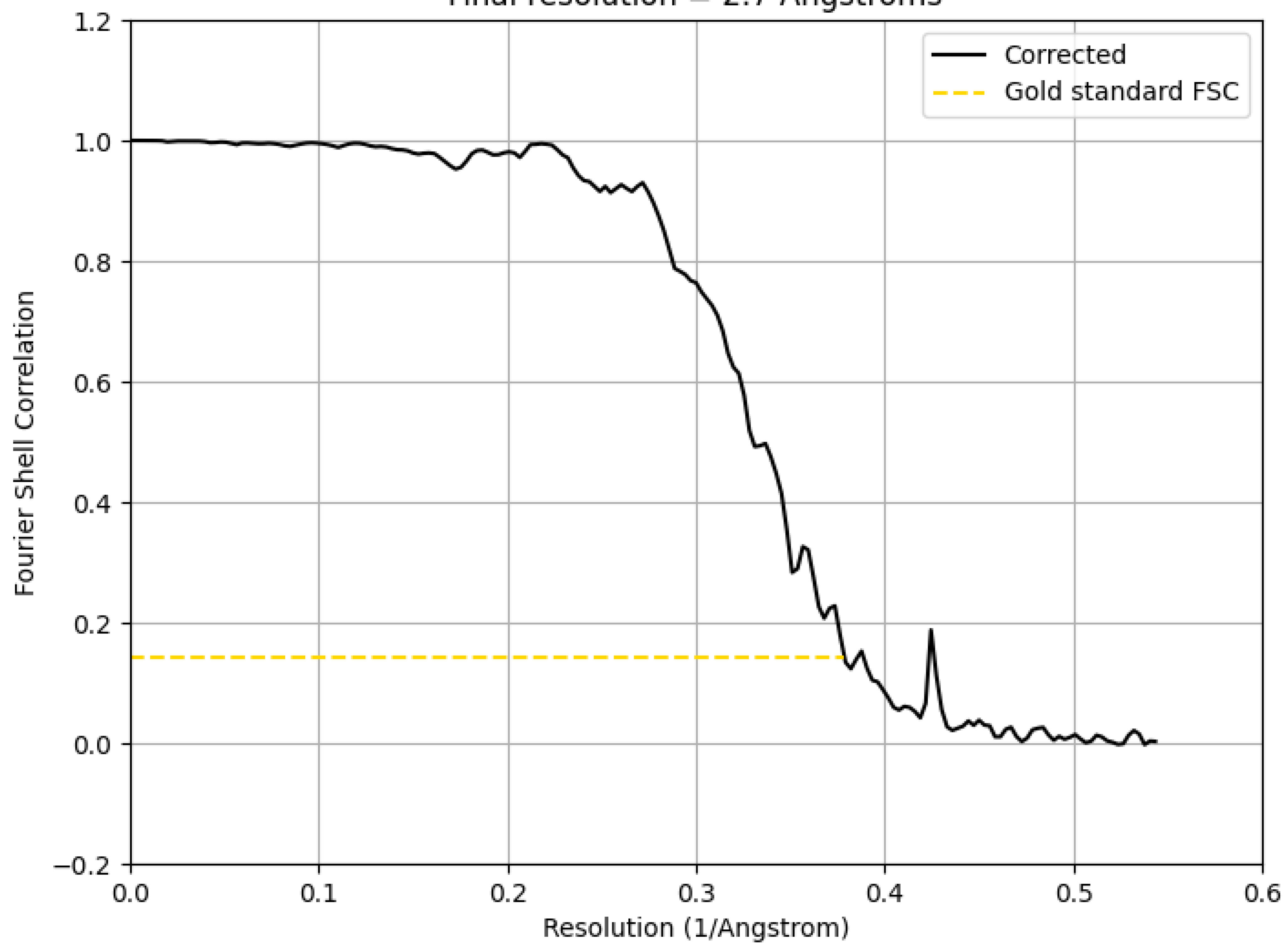**B**

Final resolution = 3.7 Angstroms

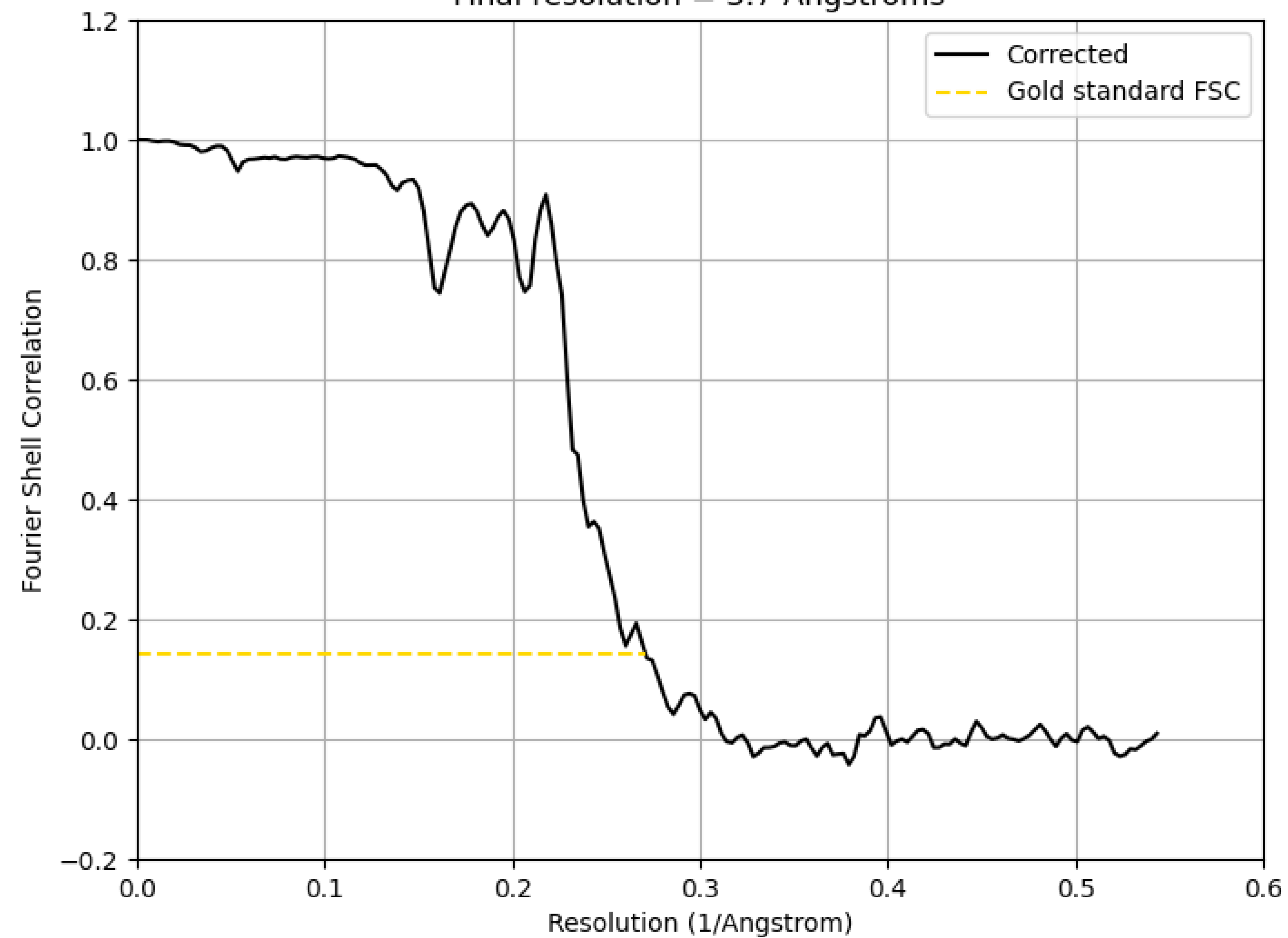

Supplement: Supplementary file 4 — Fig S4. Fourier Shell Correlation (FSC) curves for 2PF WT (A) and 2PF K58N (B). [file MDS-40-2732-s004.pdf]

# Supplementary Figure - Processing Overview K58N

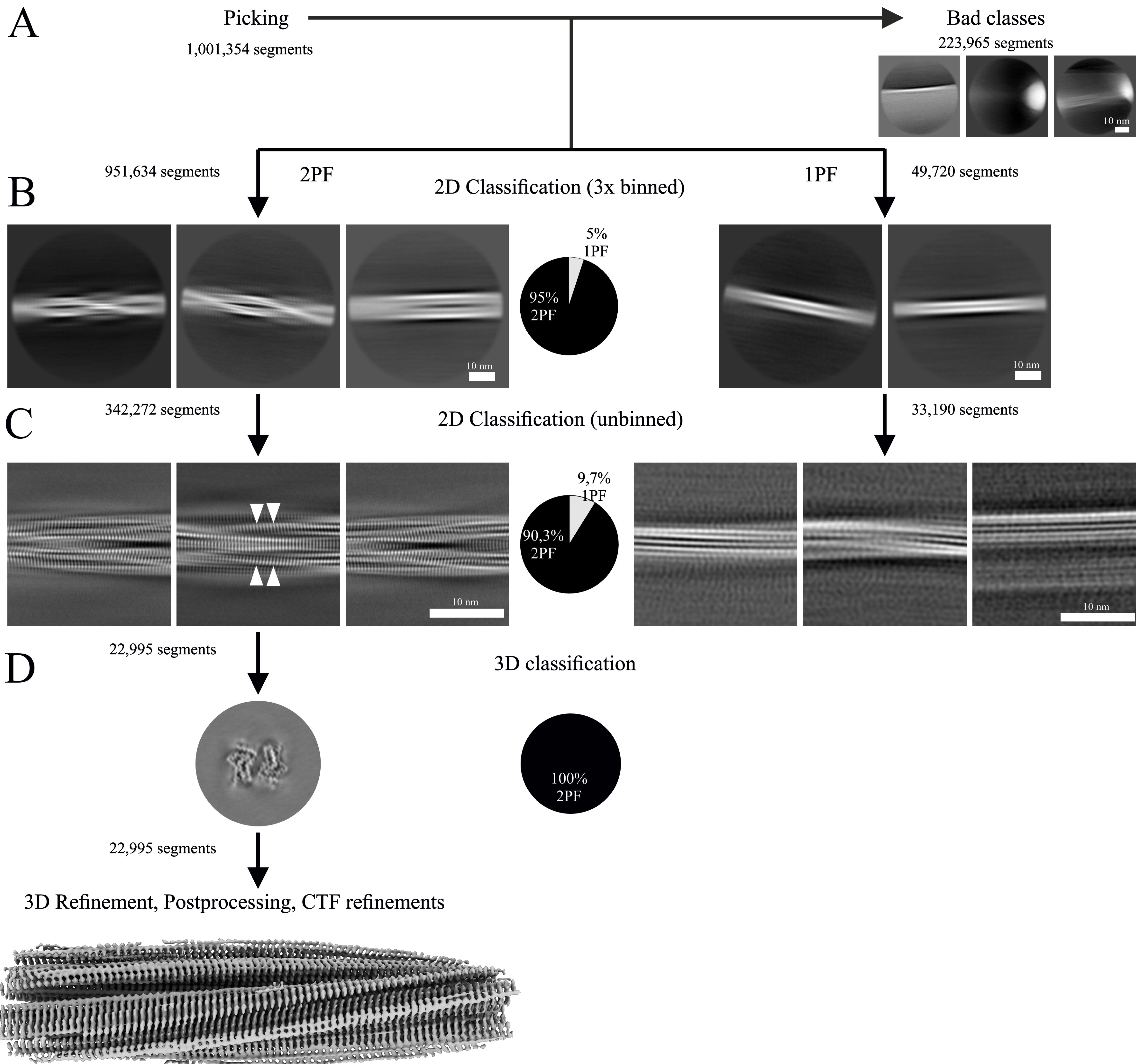

Supplement: Supplementary file 5 — Fig S5. Detailed Processing Workflow for the K58N aSyn Dataset. (A) A subset of the initially picked helical segments was removed due to artifacts, such as those containing carbon edges. The remaining segments were divided into two categories based on their structural features: segments showing two protofilaments (2PF, “wide”) and those showing a single protofilament (1PF, “narrow”). (B) During the initial classification step with three times binned segments, a significant majority of the segments were categorized as 2PF, with only a small fraction classified as 1PF, as illustrated by the provided pie chart. (C) Subsequent classification with unbinned data supported the initial results, as depicted by the adjacent pie chart. In contrast to the 2PF dataset, no clear beta‐sheet separation was observed in the unbinned class averages for the 1PF dataset, hinting at the lower quality and quantity of the 1PF segments. (D) The results of the three‐dimensional classification are presented. For the 2PF data, the refined electron density map is shown after 3D refinement, postprocessing, and CTF refinement. Due to the poor quality of particles and class averages in the 1PF dataset, further processing via 3D classification was not feasible. [file MDS-40-2732-s007.pdf]

**A**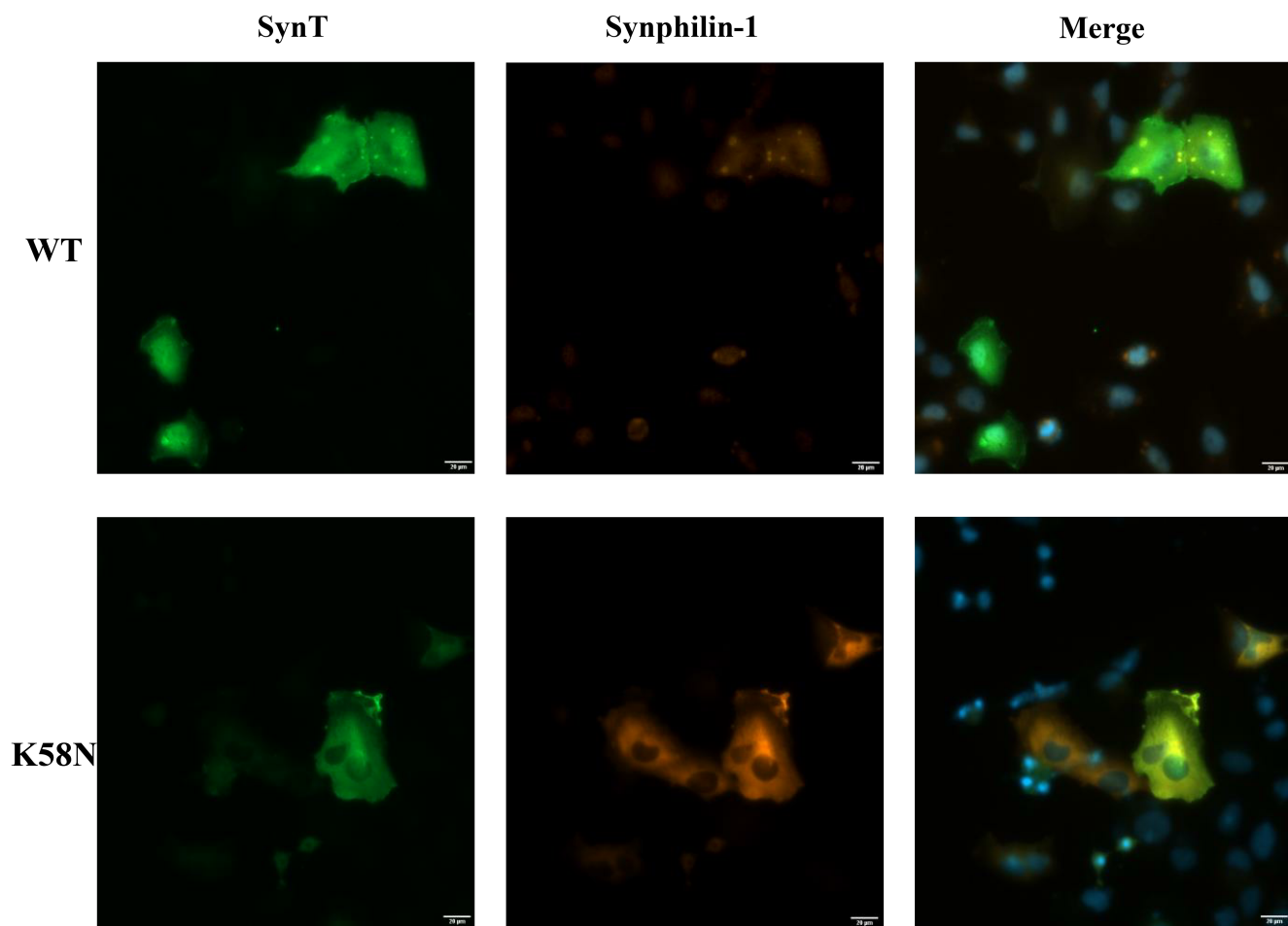**B**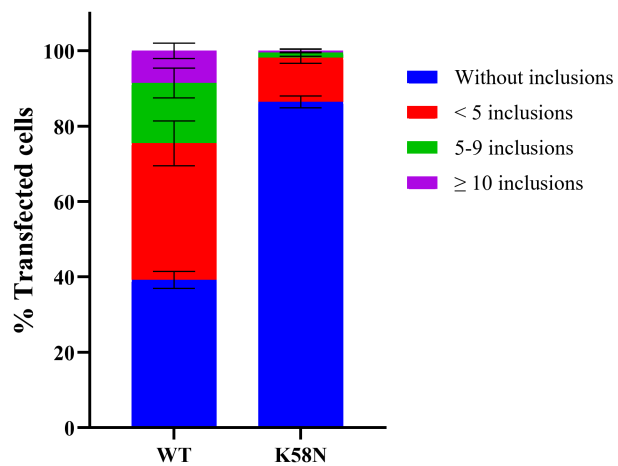**C**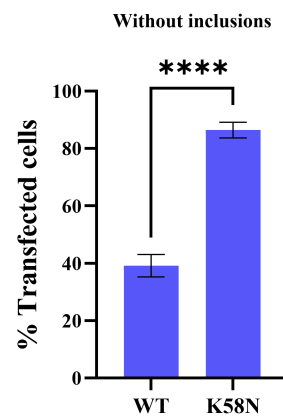

Supplement: Supplementary file 6 — Fig S6. Effect of K58N mutation on inclusion formation in cells. (A) Representative immunohistochemistry images of H4 cells showing the patterns of inclusion formation for both WT and K58N SynT variants. Scale bar: 20 μm. (B) and (C) show the quantification of the number of inclusions from transfected cells. A total of 50 cells were counted using a 20x objective for each experiment, and classified into four groups based on their inclusion patterns. Student's t‐test was used to analyze the data, and the results are shown as mean ± SD from 3 independent experiments. [file MDS-40-2732-s006.pdf]

**A**

EGFP

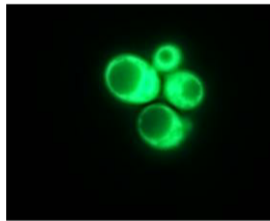

WT

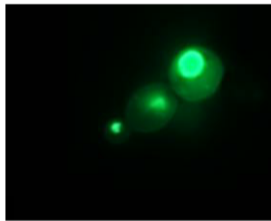

K58N

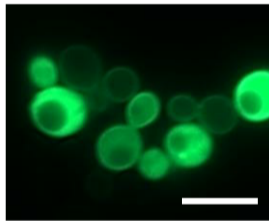**B** $10^{-1}$  $10^{-4}$ 

EGFP

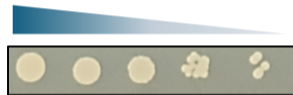

WT

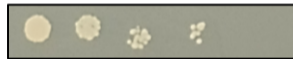

K58N

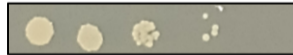

Supplement: Supplementary file 7 — Fig S7. Effect of K58N mutation on yeast cells. S. cerevisiae cells harboring WT aSyn or K58N aSyn mutation were grown to mid‐log phase. (A) aSyn localization was analyzed by fluorescence microscopy. (B) Cellular viability was evaluated by spotting assay, where cultures were spotted on SD‐URA agar plates. [file MDS-40-2732-s008.pdf]
