# Supplementary material for: A Novel α‐Synuclein K58N Missense Variant in a Patient with Parkinson's Disease
Source: Mov Disord. 2025 Sep 4;40(12):2732–45. doi: 10.1002/mds.70030 (PMC12710137; doi:10.1002/mds.70030)
Supplement: Supplementary file 8 — Supplementary Information. [file MDS-40-2732-s005.docx]

**Supplementary Information**

**Extended methods**

**Genetic testing**

Whole exome sequencing (WES) was performed at the Institute of Human Genetics at the Technical University of Munich, Germany. Genomic DNA was extracted from peripheral blood according to standard protocols. In-solution enrichment of targeted regions was accomplished using the Sure Select Human All Exon Kit (Agilent 60mb V6) and followed by paired-end sequencing of 100 base pare (bp) long reads with the Illumina NovaSeq6000 system (Illumina, San Diego, California). Average exome coverage was 102x, with 97% of the target regions being covered at least 20x. The whole *SNCA* region was covered >20x (average depth 80x). The variant c.174G>C, p.K58N was confirmed in an independent blood sample by Sanger sequencing by the Human Genetics Department at the University Medical Center Göttingen.

**Expression and purification of recombinant WT and K58N aSyn**

The p.K58N variant (NP_000336.1) was incorporated into the bacterial plasmid encoding aSyn using through site-directed mutagenesis (QuikChange II, Agilent), and Sanger sequencing was used to validate the presence of the mutation. The production of recombinant proteins was performed in BL21(DE3) *E. coli* cells that were transformed with pET21A vectors encoding either (wild type) WT or K58N aSyn, following established protocols ^1^. After extracting proteins from bacterial pellets, they were purified through sequential anion-exchange and size-exclusion chromatography (SEC) for use in aggregation assays, nuclear magnetic resonance (NMR) analysis, and lipid binding experiments. The purified proteins were concentrated in PBS buffer (pH 7.4), sterile-filtered, and stored at -80°C until further use. For NMR studies, the SEC buffer was replaced with 100 mM NaCl, 50 mM HEPES, pH 7.4. Determination of proteins concentration was done by measuring absorbance at 280 nm, applying an extinction coefficient of 5,960 M⁻¹cm⁻¹.

**Liposome preparation**

5 mg of 1,2-Dioleoyl-sn-glycero-3-phosphoethanolamine (DOPE):1,2-dioleoyl-sn-glycero-3-phospho-L-serine (DOPS):1,2-dioleoyl-sn-glycero-3-phosphocholine (DOPC) 5:3:2 w/w (Avanti Polar Lipids) were resuspended in 0.8 mL methanol:chloroform 1:1, evaporated under a nitrogen stream, lyophilized o/n and resuspended in 0.52 mL of HEPES buffer (50 mM HEPES, 100 mM NaCl, pH 7.4, 0.02% NaN_3_). The resulting turbid sample was sonicated until transparency (15 minutes, 10’’ on, 20’’ off).

**Circular dichroism (CD) spectroscopy**

Individual WT and K58N αSyn-to-lipid ratios were pipetted (ratio: 1:10, 1:20, 1:40, 1:100 and 1:200) at a protein concentration of 50 µM and the respective lipid concentrations from a 12.5 mM liposome stock in HEPES buffer (50 mM HEPES, 100 mM NaCl, pH 7.4, 0.02% NaN_3_) to a total volume of 12 µL. For CD measurement the sample was diluted in 48 µL deionized water and transferred to a 0.02 cm pathlength FireflySci cuvette for a final protein concentration of 10 µM. CD data were collected from 190 to 260 nm by using a Chirascan-plus qCD spectrometer (Applied Photophysics, Randalls Rd, Leatherhead, UK) at 20 °C, 1.5 time-per-point (s) in 1 nm steps. The datasets were averaged from three repeats. All spectra were baseline corrected against buffer in deionized water and smoothened (window size: 8).

**NMR spectroscopy**

NMR experiments were measured on a Bruker 900 MHz spectrometer equipped with a 5 mm triple-resonance, pulsed-field z-gradient cryoprobe. Two-dimensional ^1^H,^15^N and ^1^H,^13^C heteronuclear single quantum coherence (HSQC) and ^1^H,^1^H total correlation spectroscopy (TOCSY) experiments were acquired for monomer characterization at 15 °C. All experiments were performed in HEPES buffer (50 mM HEPES, 100 mM NaCl, pH 7.4, 0.02% NaN_3_) with 5 % (v/v) D_2_O. Spectra were processed with TopSpin 3.6.1 (Bruker) and analyzed using Sparky 3.115 (T. D. Goddard and D. G. Kneller, SPARKY 3, University of California, San Francisco). The combined HN/N chemical shift perturbation was calculated according to (((δHN)^2^+ (δN/10)^2^)/2)^1/2^. Secondary structure was calculated subjecting the experimental HA, CA, HN and N chemical shifts to TALOS-N ^2^.

**Molecular dynamics simulations**

Starting structures of the αSyn WT and K58N peptides (residues 51-65) were built in the PyMOL Molecular Graphics System (Version 1.8.4.0, Schrödinger, LLC). Initially, the peptides were equilibrated in a water box with 50,000 steps of energy minimization. To further equilibrate the system, 100 ps each of volume (NVT) and pressure (NPT) equilibration were performed without position restrains. The Molecular dynamics (MD) simulations were carried out in GROMACS (version 2018.3) using the AMBER99SB-ILDN force field and the TIP3P water model at a temperature of 300 K, 1 bar of pressure and with a coupling time (ζT) of 0.1 ps. The peptides were solvated in water with 150 mM NaCl, ensuring overall charge neutrality. The particle mesh Ewald algorithm was used for calculation of the electrostatic term, with a radius of 16 Å for the grid-spacing and Fast Fourier Transform. The cut-off algorithm was applied for the non-coulombic potential with a radius of 10 Å. The LINCS algorithm was used to contain bonds and angles. MD simulations were performed during 100 ns in 2 fs steps and saving the coordinates of the system every 10 ps. The α-helical content over the simulation trajectory was analyzed using the PyMOL Molecular Graphics System. Error bars were calculated from the results of 40 peptides (5 peptides in the water box of 8 independent simulations).

**In vitro thioflavin T fluorescence-based aggregation assays**

For the thioflavin T (ThT) aggregation kinetics assay setup, lyophilized protein was reconstituted in sterile filtered bidistilled water. To get rid of any potential aggregates in protein solutions, samples were first centrifuged at 14,000 rpm for 5 minutes in 100 kDa MWCO filter tubes (Sigma-Aldrich, MO, USA) to collect the filtrate containing monomeric aSyn. Protein concentration was determined on an LVis Plate (BMG Labtech; Ortenberg, Germany) using a CLARIOstar Plus plate reader (BMG Labtech; Ortenberg, Germany) employing the previously mentioned extinction coefficient for aSyn. Prior to initiating the assay, a master mix of 0.5 mg/mL WT or K58N aSyn was prepared in 150 mM NaCl, 10 mM PBS (pH 7.4), 1 mM EDTA, 0.002% SDS, and 25 µM ThT, with 100 µL added into each well in quadruplicate per condition, in addition to the use of protein-free master mix as a blank. The aggregation assay was conducted using CLARIOstar Plus plate reader (BMG Labtech, Ortenberg, Germany) and Costar black, clear-bottom 96-well half-area plates with preloading a single 1-mm glass bead to each well. Plates were sealed with microplate tape and transferred to the reader, with the following settings: orbital shaking (60 seconds on, 30 seconds off) at 400 rpm, 37 °C, in 3.66 min cycles for a total of 1,000 cycles. ThT fluorescence was measured at the end of each cycle with bottom optics, excitation at 450 ± 10 nm, and emission at 480 ± 10 nm. Aggregation curves were then blank-corrected and normalized to the maximum fluorescence for each run.

**Cryogenic electron microscopy (cryo-EM) imaging and analysis of WT and K58N fibrils**

Cryo-EM sample Preparation

Copper 200 mesh R2/1 Quantifoil grids were plasma cleaned for 45 seconds at medium power using a Harrick Plasma PDC-32G-2 plasma cleaner and then mounted on a Vitrobot Mark IV (Thermo Fisher). aSyn fibrils, prepared at a concentration of 0.5 mg/mL in 30 mM Tris-HCl buffer at pH 7.5, were sonicated in a waterbath sonicator for 10 minutes on medium settings with a 0.5s ON/OFF interval. Shortly thereafter a 3 mL aliquot of the fibrils was applied to the grid. Blotting was performed with a blot force of 7 for 5 seconds. During the freezing process, the Vitrobot chamber was kept at 10°C with 100% humidity.

Data Collection

Cryo-EM datasets were acquired using a Titan Krios electron microscope (Thermo Fisher) equipped with a Falcon4i detector operated in counting mode. For this study, we collected a dataset for the K58N aSyn mutant under the described conditions and used a previously acquired WT dataset, originally collected as part of the G14R study, as a control. Both datasets were collected at a nominal magnification of 130,000x, yielding a pixel size of 0.92 Å. The K58N dataset consisted of 5,813 movies, while the WT dataset comprised 4,092 movies. The defocus range for both datasets was maintained between -1.2 and -2.4 µm. A total electron dose of 40 e⁻/Å² was applied, with exposures of 6.59 e⁻/Å²*s for the WT dataset and 6.61 e⁻/Å²*s for the K58N dataset. An energy filter with a 15 eV slit width was employed during data collection.

Data Processing

The raw EER movies were fractionated, aligned, and summed using MotionCor2 with a dose per frame of 1 e⁻/Å². Contrast transfer function (CTF) parameters were estimated using CTFFIND4. To select segments for both the WT and K58N datasets, a crYOLO model was employed, which had been trained on previously grown recombinant aSyn filaments. For this, fibrils from about 50 micrographs of a previous WT aSyn dataset were manually picked in RELION-4, and the coordinates were exported to train a picking model in crYOLO. This model was subsequently used to automatically pick fibrils across all micrographs. The filament coordinates were imported into RELION and used to extract helical segments with an inter-box spacing of approximately 15 Å, corresponding to three asymmetric units per segment. Segments were first extracted with a box size of 768 pixels, binned three times to a final box size of 256 pixels, and then subjected to 2D classification. After excluding picking artifacts, such as carbon edges, the remaining particles were used to extract unbinned segments with a box size of 384 pixels and a pixel size of 0.92 Å/px. Another round of 2D classification was performed, and only the classes showing beta-sheets were selected for further analysis. In case of 1 protofilament (PF) classes for the K58N dataset, no further classifications were carried out since class averages did not show betasheets. Segments of the selected classes underwent 3D classifications, during which helical parameters were systematically scanned as the crossover distance was not visible in the micrographs and the twist could not be determined directly. Multiple 3D classifications were carried out with a fixed helical rise of 4.75 Å and a twist ranging from -0.5° to -1.7° in roughly 0.05° increments. A featureless cylinder was used as the initial model. Classes showing distinct polypeptide signals were utilized as initial models for subsequent 3D refinements that were performed using a sampling interval of 1.8° and a T-value of 15 to 30. Postprocessed models were then used for CTF refinements and final 3D refinements to optimize resolution and model quality.

**aSyn aggregation studies in cells**

Cell culture

Human neuroglioma cells (H4) were maintained in Opti-MEM I Reduced Serum Medium (Life Technologies- Gibco, Carlsbad CA, USA) supplemented with 10% Fetal Bovine Serum Gold (FBS) (PAA, Cölbe, Germany) and 1% Penicillin-Streptomycin (PAN, Aidenbach, Germany). The cells were grown at 37ºC in an atmosphere of 5% CO2.

Cell Transfection

Twenty-four hours prior to transfection, approximately 80000 H4 cells were plated per well in a 12-well plate (Costar, Corning, New York, USA). Six hours prior to transfection, the medium was replaced with a fresh one. Transfection protocol using Fugene methodology was carried out as described by the manufacturer. Briefly, ratio of 1 (equal amounts of the plasmids encoding SynT WT Kozak or SynT K58N Kozak with/without Synphilin-1-V5) :3 (Fugene solution) mix was prepared in Optimem medium without adds. The mix was incubated for 20 min and added dropwise to the cells while the plate was gently rocked.

Immunocytochemistry

Forty hours after transfection, the medium was removed, the cells were washed with PBS and fixed with 4% paraformaldehyde (PFA) for 30 minutes at room temperature (RT). Cell permeabilization with 0.1% Triton X-100 (Sigma-Aldrich, St. Louis, MO, USA) for 20 minutes at RT was performed, followed by blocking in 3% Bovine serum albumin (Nzytech, Lisbon, Portugal) in PBS 1x for 1 hour. Afterwards, cells were incubated with primary antibody mouse anti-ASYN (1:1000, BD Transduction Laboratory, New Jersey, USA) overnight and secondary antibody Alexa Fluor 488 goat anti-mouse (Life Technologies- Invitrogen, Carlsbad, CA, USA) for 2 hours at RT. Finally, cells were stained with DAPI (Carl Roth, Karlsruhe, Germany) (1:5000 in PBS 1x) for 10 minutes, and the coverslips mounted in SuperFrost® Microscope Slides treated with Mowiol (Calbiochem, San Diego, CA) dried and stored at room temperature until further visualization and analysis. Images were acquired using a confocal point-scanning microscope (Zeiss LSM 900 with Airyscan, Carl Zeiss, Jena, Germany). For each condition, 50 images of the 10 slices Z-Stack were taken using the 63x objective (Objective Plan-Apochromat 63x/1.4 Oil DIC M27) and specific definitions for each staining in the ZEN Software (Carl Zeiss, Jena, Germany).To quantify the number of aggregates and the size of the aggregates (area of the inclusions) inside the cells, the aSyn channel was selected and the images were thresholded The images were then analysed using the Analyse particle plugin from Fiji open-source software ^3^.

**Solubility and dynamic pS129 reversibility experiments**

Plasmids and lentivirus production: synthetic cDNA sequences encoding WT or K58N aSyn were digested at the SpeI/NotI enzymes restriction sites and then ligated into pLVX-EF1a-IRES ZsGreen1 vector (TaKaRa) for their expression, driven by the EF1a promoter. Lentiviral packaging was carried out in 293-T cells as previously described ^4^. Briefly, 293-T cells were transfected with plasmids encoding WT or K58N aSyn along with the packaging plasmids pMD2.G and psPAX2 (Addgene #12259 and #12260). After transfection, the viral particles from the culture supernatant were collected, and subjected to ultracentrifugation at 100,000g for obtaining purified/concentrated viral particles. The purified viral pellet then reconstituted in neurobasal medium containing B-27 and Glutamax (Gibco), resulting in a yield around 2·5x10^6^ viral particles per μL. To investigate the impact of K58N mutation on phosphorylation status of aSyn at S129, we cultured primary cortical neurons from E18 pregnant *SNCA* knockout (*SNCA*−/−) rats on 24‐well plates previously coated with poly‐d‐lysine, and induced the expression of human WT or K58N proteins by lentiviral transduction at DIV5 as described ^5^. To assess the solubility of aSyn of K58N vs. WT aSyn, we performed sequential protein extraction to isolate cytosol (C) vs. membrane (M) protein fractions using the on-plate extraction technique as previously described ^6^. For the experiments assessing dynamic reversibility of pS129, cortical cultures were treated at DIV17-21 with vehicle (DMSO), 20 µM picrotoxin (PTX), 1µM tetrodotoxin (TTX), or combination of both PTX and TTX. Following treatment for 2h or 4h intervals, cell lysis and immunoblotting was conducted to measure the levels of total and pS129 aSyn as previously described ^4,5^. For this study, at least three independent experiments were carried out on different days, with a total of 10-16 biological replicates. Data presented in Fig. 6 B, D, and I-L are statistically analyzed by an unpaired *t*‐test with Welch's correction, while data in F-G were analyzed by Brown‐Forsythe and Welch ANOVA with Dunnett's T3 *post hoc* test for multiple comparisons. Data in Fig. 6 H were analyzed by a two-way ANOVA with Šídák's multiple comparisons test. Please note: In the experiments assessing the solubility and dynamic reversibility of pS129, both G14R ^7^ and G58N mutants were characterized in parallel. Consequently, the data points for WT aSyn are identical across both sets of experiments (Fig. 6 D, F, H, I, J, K, and L).

**aSyn expression and purification for phase separation studies**

Recombinant WT and K58N human full-length aSyn was expressed in BL21(DE3) competent Escherichia coli (C2527, NEB, Ipswich, US) using vector pET28a (Addgene #178032). Bacteria were cultured in LB media supplemented with 50 μg/mL kanamycin (37 °C, constant shaking at 250 rpm). Expression was induced at an OD600 of 0.8 using 1mM isopropyl β-D-1-thiogalactopyranoside (IPTG) and cultured overnight at 25 °C. Cell pellets were harvested by centrifugation at 4000 × g for 30 minutes (AVANTI J-26, Beckman Coulter, USA). aSyn was purified using a protocol previously described (2). Briefly, the cell pellet was resuspended in lysis buffer (10 mM Tris, 1 mM EDTA, Roche cOmplete EDTA free protease inhibitor cocktail, pH 8). The cells were disrupted using a cell disruptor (Constant Systems, Daventry, UK) and were ultracentrifuged at 4 °C, 186,000 × g for 20 minutes (Ti-45 rotor, Optima XPN 90, Beckman Coulter, USA). The supernatant was collected and heated for 20 minutes at 70 °C to precipitate heat-sensitive proteins, followed by ultracentrifugation as above. Streptomycin sulfate (5711, EMD Millipore, Darmstadt, Germany) was added at a final concentration of 10 mg/mL to the supernatant and continuously stirred at 4 °C for 15 minutes to precipitate DNA, followed by ultracentrifugation as above. Ammonium sulfate (434380010, Thermo Scientific) was added at a final concentration of 360 mg/mL to the supernatant and continuously stirred at 4 °C for 30 minutes to precipitate the protein. The precipitated protein was then centrifuged at 500 × g for 15 min, dissolved in 25 mM Tris, pH 7.7, and dialyzed overnight against the same buffer to remove salts. The protein was purified using ion exchange on a HiTrap^TM^Q HP 5mL anion exchange column (17115401, Cytiva, Sweden) using gradient elution with 0-1M NaCl in 25 mM Tris, pH 7.7. The collected protein fractions were run on SDS-PAGE and pooled fractions were further purified using size-exclusion chromatography on a HiLoad^TM^ 16/600 Superdex^TM^ 75 pg column (28989333, Cytiva, Sweden). The fractions were collected, and their purity was confirmed using SDS-PAGE analysis. Protein concentrations were determined by measuring absorbance at 280 nm using an extinction coefficient of 5,600 M−1cm−1. The monomeric protein was frozen in liquid nitrogen and stored in 25 mM HEPES buffer pH 7.4 at -70 °C. pET28a Cdk2ap1CAN was a gift from Lin He (Addgene plasmid # 178032; http://n2t.net/addgene:178032; RRID:Addgene 178032) (3).

**aSyn labelling**

Labelling of aSyn was performed in bicarbonate buffer (C3041, Sigma) at pH 8 using NHS-ester active fluorescent dye AlexaFluor 488 5-SDP ester (A30052, Invitrogen Thermo Fisher). Excess-free dye was removed by buffer exchange using PD10 desalting columns (IP-0107-Z050.0-001, emp BIOTECH, Generon). Labelled protein concentrations were estimated using the molar extinction coefficient ε494 nm = 72,000 M^−1^cm^−1^.

**aSyn phase separation assays**

All aSyn phase separation assays were performed in 25 mM HEPES, pH 7.4 unless mentioned otherwise. Phase separation was induced by mixing aSyn and PEG-8000 (BP223, Fisher Bioreagent) in the presence of calcium (21108, Sigma) as indicated. Images for phase-separated samples were acquired on an LSM780 confocal microscope (Zeiss, Oberkochen, Germany) using a 63x oil immersion objective. Zen 2.3 (black edition) and Zen 2.6 (blue edition) were used for data collection and image export. Images were taken at the indicated aSyn concentration, where aSyn was supplemented with 1% Alexa 488 labelled aSyn. For turbidity measurements phase separation samples were set up as described above using indicated concentrations of aSyn and PEG-8000 in the presence of calcium (21108, Sigma). The turbidity of the samples was measured at 350 nm, 25 °C using 96-well Greiner optical bottom plates on a CLARIOstar plate reader (BMG LABTECH, Ortenberg, Germany) under quiescent conditions. CLARIOStar 5.01 was used for data acquisition. A sample volume of 100 μL was used, and readings were taken within 5 minutes of sample preparation. Raw turbidity data are plotted with background subtraction using GraphPad Prism 9.3.1. Data were obtained from four independent repeats.

**Plasmids**

WT human full-length *SNCA* and VAMP2, encoding aSyn and VAMP2, were cloned from cDNA obtained from human neuroblastoma cells (SH-SY5Y) and inserted into the pEYFP-N1 and pMD2.G vector (Addgene #96808, #12259) with a C-terminal YFP and Flag-tag, respectively. aSyn K58N was generated using KLD substitution (M0554S, NEB, Ipswich, US). All sequences were verified by sequencing. 5HT6-YFP-Inpp5e was a gift from T. Inoue (Addgene plasmid 96808; RRID: Addgene_[96808](http://n2t.net/addgene:96808))[128](https://www.nature.com/articles/s41556-024-01451-6#ref-CR128). pMD2.G was a gift from D.Trono (Addgene plasmid 12259; RRID: Addgene_[12259](http://n2t.net/addgene:12259)).

**Cell culture and transfection**

HeLa cells were obtained from the European Collection of Cell Cultures (ECACC 93021013) and grown in Dulbecco’s modified Eagle’s Medium (DMEM) high glucose (31966-021, Gibco) supplemented with 10% fetal bovine serum (FBS, F7524, Sigma) and 1% Penicillin/Streptomycin (P0781, Sigma). Cells were grown at 37 °C in a humidified incubator with 5% CO2. Cells were tested for mycoplasma contamination using MycoStrip^TM^ (IvivoGen, Toulouse, France). Cells were plated at 20,000 cells/well in 8-well ibidi dishes (80807, ibidi, Gräfelfing, Germany) for confocal imaging or in 48-well plates (Cellstar, 677 180, Greiner bio-one) for incuCyte experiments. Cells were transfected the following day using Fugene HD Transfection reagent according to the manufacturer’s protocol (E2311, Promega). Briefly, per reaction 12.5 μL OptiMEM (31985-062, Gibco) were set up in 1.5 mL sterile Eppendorf tubes. A total of 250 ng of DNA and 0.75 μL of Fugene reagent were added and incubated for 15 min at room temperature. The transfection mix was added to the cells for 1 min and then topped up with 300 μL complete media. Cells were imaged the next day.

**Confocal microscopy and IncuCyte**

Live cell confocal imaging was performed on an LSM780 microscope (Zeiss, Oberkochen, Germany) using a 63x oil immersion objective. YFP fluorescence was excited with the 514 laser at 2% laser power. Zen 2.3 (black edition) and Zen 2.6 (blue edition) were used for data collection and image export. For quantitative evaluation of condensate formation cells were imaged with the IncuCyte S3 (Essen BioScience, Newark, UK). Phase brightfield and green fluorescence images were taken using a 20x objective at a 4-hour interval at 200 ms exposure, condensate formation (% of cells showing condensate formation) was evaluated 16 hours after transfection. IncuCyte 2021A was used for data analysis. At least three biological repeats with three technical repeats each were analysed blinded to the investigator.

**Quantification and statistical analysis**

Data analysis and statistical analysis was performed using Excel 2016 and GraphPad Prism 9.3.1. All data are represented as mean ± standard error (SD) if not indicated otherwise. Statistical analysis was carried out using unpaired two-tailed t-test. Statistical parameters are reported in the Fig.s and the corresponding Fig. Legends. Exact p-values are shown. Data distribution was assumed to be normal but this was not formally tested. No statistical methods were used to pre-determine sample sizes but our sample sizes are similar to those reported in previous publications (4–6). Samples were randomly allocated into experimental groups. Data collection and analysis have been performed blinded when indicated. Data were included if the control (wild-type) showed appropriate condensate formation.

**Yeast Plasmids**

The aSyn-K58N variant was constructed by site-directed mutagenesis using the QuickChange II Site-Directed Mutagenesis Kit (Agilent Technologies, SC, USA), in the plasmid backbone p426GPD encoding the WT aSyn-GFP, following the manufacturer’s instructions. We also constructed a plasmid expressing only GFP as a control, by introducing the GFP coding sequence as a SpeI-XhoI digested PCR product in the p426GPD backbone. All constructs were confirmed by DNA sequencing.

**Yeast cell growth conditions, viability assay and fluorescence microscopy**

The Saccharomyces cerevisiae yeast strain BY4741 (MATa his3Δ1 leu2Δ0 met15Δ0 ura3Δ0) was transformed with GFP, WT aSyn and K58N plasmids by standard lithium acetate method. All strains were grown overnight at 30 °C 180 rpm in yeast minimal synthetic defined (SD) medium (Takara Bio), supplemented with a drop-out mix (Takara Bio) lacking the amino acid uracil (SD-URA) for transformant selection, at a volume/medium ratio of 5:1. The assessment of cellular viability was achieved by spotting assay. Here, cultures grown to mid-log phase were standardized to equal cellular densities, serially diluted 10-fold starting with an OD600nm of 1 and spotted on SD-URA agar plates. Following 3 days incubation at 30 °C the plates were photographed. aSyn cellular localization was evaluated by fluorescent microscopy. Images were attained with an epifluorescence microscope Zeiss Axio Observer equipped with a 100x oil objective lens.

**Supplementary references**

1. Al-Azzani M, König A, Outeiro TF. Production of recombinant alpha-synuclein: still no standardized protocol in sight. Biomolecules. 2022;12:324.
2. Shen Y, Bax A. Protein backbone and sidechain torsion angles predicted from NMR chemical shifts using artificial neural networks. J Biomol NMR. 2013;56:227–241.
3. Schindelin J, Arganda-Carreras I, Frise E, et al. Fiji: an open-source platform for biological-image analysis. Nat Methods. 2012;9:676–682.
4. Ramalingam N, Dettmer U. Temperature is a key determinant of alpha- and beta-synuclein membrane interactions in neurons. J Biol Chem. 2021;296:100271.
5. Ramalingam N, Brontesi L, Jin S, Selkoe DJ, Dettmer U. Dynamic physiological α-synuclein S129 phosphorylation is driven by neuronal activity. NPJ Parkinsons Dis. 2023;9:4.
6. Ramalingam N, Brontesi L, Jin S, Selkoe DJ, Dettmer U. Dynamic reversibility of α‐synuclein serine‐129 phosphorylation is impaired in synucleinopathy models. EMBO Rep. 2023;24:e56789.
7. Brücke C, Lázaro DF, Ramalingam N, et al. A novel alpha-synuclein G14R missense variant is associated with atypical neuropathological features. medRxiv. 2024. <https://doi.org/10.1101/2024.09.23.24313864>
